# Actogram analysis of free-flying migratory birds: new perspectives based on acceleration logging

**DOI:** 10.1007/s00359-017-1165-9

**Published:** 2017-03-25

**Authors:** Johan Bäckman, Arne Andersson, Lykke Pedersen, Sissel Sjöberg, Anders P. Tøttrup, Thomas Alerstam

**Affiliations:** 1 Department of Biology, Lund University, Ecology Building, 22362 Lund, Sweden; 2 Center for Macroecology, Evolution and Climate, Natural History Museum of Denmark, University of Copenhagen, Universitetsparken 15, 2100 Copenhagen, Denmark

**Keywords:** Accelerometer, Bird migration, Flight pattern, Activity, Annual cycle

## Abstract

The use of accelerometers has become an important part of biologging techniques for large-sized birds with accelerometer data providing information about flight mode, wing-beat pattern, behaviour and energy expenditure. Such data show that birds using much energy-saving soaring/gliding flight like frigatebirds and swifts can stay airborne without landing for several months. Successful accelerometer studies have recently been conducted also for free-flying small songbirds during their entire annual cycle. Here we review the principles and possibilities for accelerometer studies in bird migration. We use the first annual actograms (for red-backed shrike *Lanius collurio*) to explore new analyses and insights that become possible with accelerometer data. Actogram data allow precise estimates of numbers of flights, flight durations as well as departure/landing times during the annual cycle. Annual and diurnal rhythms of migratory flights, as well as prolonged nocturnal flights across desert barriers are illustrated. The shifting balance between flight, rest and different intensities of activity throughout the year as revealed by actogram data can be used to analyse exertion levels during different phases of the life cycle. Accelerometer recording of the annual activity patterns of individual birds will open up a new dimension in bird migration research.

## Introduction

New tracking technologies, like satellite tracking including GPS data for larger birds and light-based geolocator tracking for smaller birds, have revolutionised the study of bird migration (Newton 2008; McKinnon et al. 2013). This on-going revolution also involves the use of miniaturised electronic sensors for biologging of internal factors (related to, e.g. the bird’s activity, behaviour, physiology and orientation) and external factors (besides light level also ambient temperature, barometric pressure, aerodynamic data, etc.) for migratory individuals. Logging of accelerometer data has been used for three main purposes in studies of animal behaviour—(1) for classifying behaviour and mode of locomotion and flight, (2) for estimating energy demands (these two aspects have been reviewed by Brown et al. 2013) and, most recently, (3) for recording actograms of individuals in order to analyse daily and annual activity and flight patterns in bird migration. The latter has recently been made possible for small migratory birds (Liechti et al. 2013; Bäckman et al. 2016; Hedenström et al. 2016).

In this contribution, we give a brief background about the principles and use of accelerometry, particularly about accelerometer devices for small birds, in “Accelerometer measurements and individual activity patterns in bird migration” section. Our focus is accelerometer studies in the field of bird migration, and for a more general review of accelerometer studies of animal behaviour and energetics, we refer to Brown et al. (2013). We then proceed to summarise and illustrate how new insights about flight patterns (“Migratory flights—when to fly and when to stop?” section) and about rhythms of rest and activity (“Rhythms of rest and activity throughout the year” section) among free-flying migratory birds will become possible based on analyses of accelerometer and actogram data. This technology is still in its infancy and only a few accelerometer studies of migratory birds have been published up to now. However, we expect a strong increase in the use of this technique in bird migration studies in the near future. Hence, in addition to reviewing the existing accelerometer studies of migrating birds, we will use the very first annual actograms for two individuals of free-flying nocturnal passerine migrants (red-backed shrike *Lanius collurio*) to explore and illustrate in a heuristic way different questions that can be addressed by this new type of movement data. Needless to say, the patterns emerging for only two individuals will serve only as examples and illustrations, and conclusion about the birds’ general behaviour must await analyses based on accelerometer data from large samples of individuals. The results presented in this paper are from a red-backed shrike that was recaptured in 2016, but also include some comparisons with data from a bird recaptured in 2015 that have been presented in Bäckman et al. (2016). Both of these birds were equipped with loggers in 2014 that measured activity during 2014–2015.

Thus, our contribution is a combination of a review of accelerometer studies in bird migration and a first exploration of possibilities provided by analyses of actogram data for free-living migratory songbirds.

## Accelerometer measurements and individual activity patterns in bird migration

### Acceleration and accelerometers

Acceleration is the rate of change in velocity. Devices that measure acceleration—accelerometers—are often small micro electro-mechanical systems (MEMS), consisting of a small mass suspended in a flexible beam. Acceleration will deflect the mass from its resting position and the deflection is measured and usually converted to a digital signal which is easily interfaced to, for example, a microcontroller. Sensors are manufactured in 1–3 axes units that measure in one, two or all three dimensions (Dadafshar 2014).

Cell phones, game consoles and automotive applications have driven development of accelerometer (and other) sensors to MEMS-type devices that are very small in size and consume very little power when operating—a necessity in portable electronics (Bhushan 2010) including small accelerometer devices that can be attached to animals. An accelerometer attached to an animal will detect physical activity and can even be used to estimate energy expenditure (Wilson et al. 2006; Zeng and Zhao 2011). However, an accelerometer does not directly measure displacement, but rather change of speed and/or direction. Velocity is acceleration integrated over a time interval, and displacement is the integral of velocity, so it is theoretically possible to track an animal with an accelerometer (“dead reckoning”). However, sampling errors, though small, will accumulate and often lead to unacceptable precision over time. The method works reasonably well for marine animals in still water (Wilson et al. 2007), possibly for terrestrial movement (Bidder et al. 2015) but dead reckoning is difficult to use for flying animals, where the surrounding air usually moves with speeds of the same order as the animals.

### Accelerometry used to characterise animal behaviour

Static acceleration due to gravity will be detected by an accelerometer and be superimposed on any dynamic acceleration caused by movement. The gravity vector can reveal body posture if the accelerometer device is attached in a fixed way to the animal, where the best estimate is achieved with a multidimensional accelerometer (Kemp et al. 1998; Yoda et al. 2001). An accelerometer is especially well suited to detect movement in animals where locomotion is performed with a pronounced oscillating movement, like swimming or flying animals (birds, bats). The approximate frequency and magnitude of oscillation is often known, and this allows us to configure an accelerometer that rather easily recognises locomotion from other kinds of movement (Broell et al. 2013). Accelerometers that are used for animal movement research typically produce data with a sampling frequency in ranges from a few Hz up to hundreds of Hz (Brown et al. 2013), often from 3-axis devices. These quickly produce large volumes of data, and quite a bit of thought and effort has gone into the challenge of evaluating and interpreting accelerometer data (e.g. Sakamoto et al. 2009; Qasem et al. 2012; Collins et al. 2015). Most accelerometer-based studies on movement and behaviour of free-ranging wild animals have focused on marine mammals or (with the exception of a recent study on bats; Weller et al. 2016) larger terrestrial mammals (Brown et al. 2013). Bird species that have been studied are penguins or larger flying birds, for example, frigatebirds (Weimerskirch et al. 2016), vultures (Williams et al. 2015) cormorants (Wilson et al. 2006), geese (Bishop et al. 2015) and gulls (Shamoun-Baranes et al. 2016). In these cases accelerometers are often configured to sample 3-axis data either continuously or during rather long sequences.

Other sensors may be more suitable than an accelerometer to detect certain types of animal behaviour; for instance, a gyroscope detects rotation and is a better choice to detect and quantify rotary movements (Noda et al. 2014). Magnetometers detect geographical orientation and can be used not only to estimate direction of movement but also body posture (if inclination is known), just like the accelerometer.

### Design of accelerometer devices for small birds

There is a rule-of-thumb that a device attached to a bird should weigh 3–5% or less of the animal’s mass. This means that to study the movement of smaller migratory birds like songbirds the accelerometer device mass has to be in the order of 1 gram or less. It is often also desirable to have an operational life of at least a year, and designing an accelerometer logger with these properties is a challenge. Most of the mass will be attributed to a (still small) battery and a lesser part to electronic circuits. Size restrictions allow only a relatively small data memory. To avoid storing large data volumes, it is very helpful to perform at least part of the signal analysis of raw data onboard. A first step is to reduce the amount of raw data, and one way is to avoid routinely sampling 3D data where they are not strictly motivated. For simpler behaviour classification with a general focus on relative amount of activity and distribution of activity over time, it can be enough to sample acceleration along only one axis/dimension.

For smaller passerines, flight can be detected with very brief sampling bouts thanks to their relatively high wing-beat frequency. An example: A small passerine (of 20–30 g body mass) typically has a wing-beat frequency in the order of 12 Hz which corresponds to a ½ wing-beat cycle time of 40 ms. If a bird flies with continuous flapping, it will theoretically be enough to sample for a half wing-beat cycle in order to detect a significant deviation in acceleration from gravity. This is still true for birds that fly with bounding flight; even in in-between wing-beats, during the descending trajectory, the bird will continuously change vertical velocity. To detect continuous flight with very good certainty, one can make repeated sampling bouts with short intervals, e.g. a couple of seconds.

Furthermore, repeated and regular brief sampling of movement/no movement can be compiled into a gradual estimate of activity levels that range from continuous activity (like long-distance flights during migratory nights) to complete inactivity, like sleep. Activity levels in-between will represent foraging or breeding activities—feeding/hunting, predator escape, preening, territory defence, etc., see Bäckman et al. (2016) for an example of implementation of such a sampling scheme.

To determine body posture requires a very brief sampling. Liechti et al. (2013) studied alpine swifts *Tachymarptis melba* and used an approach of repeated sampling where dynamic acceleration was estimated every 4 min by sampling 32 values with 10 Hz frequency, saving of the mean value and sum of difference between values. A similar approach was used by Bäckman et al. (2016) studying red-backed shrikes and by Hedenström et al. (2016) studying common swifts *Apus apus*, who sampled 50 values every 5 min but with 100 Hz frequency. In these accelerometer loggers, a threshold model to judge if the bird was flying actively or not was used. In the case of common swifts, additional accelerometer measurements in a complimentary axis were made to verify if the bird was resting on the ground in an upright position or not, but only if primary measurement indicated that activity was zero or very low (Hedenström et al. 2016).

There is a great potential to investigate activity in songbirds using accelerometers if they are used in an economical way regarding energy use and data generation. Even if we restrict the sampling to “only” detect movement or not, we can still reveal rather detailed data on annual activity patterns (from continuous movement/flight to different intensities of intermittent movement to continuous inactivity) that are not possible to collect in other ways. We can estimate duration of long-distance flight events as well as stopover events, giving rather detailed information on migration time budgets. By more careful analysis of activity level variation, we can estimate relative activity at different stopover sites; it is likely that activity variation during stopover events reflects differences in foraging effort and one can speculate that this indicates that sites differ in habitat quality. Certainly, this will have to be verified using direct observations.

Accelerometer measurements combined with location data will be even more valuable when evaluating the annual scheme of a migrating passerine. Devices combining GPS and accelerometer have been developed (e.g. Bouten et al. 2013) but these are far too heavy for use on songbirds. GPS is currently difficult to implement on a sub-1g device, but by using geolocation-by-light methods it is possible to design a light-weight alternative. Flight altitude recording will require an additional sensor. Temperature decreases with altitude but temperature measurements by a body mounted datalogger are more or less influenced by heat-loss from the bird. A far better sensor would be a pressure-sensing device. Pressure data will also be useful to verify continuous flight detections from accelerometer data since nocturnally migrating passerines often fly at high altitudes.

As we will show in this contribution, accelerometer measurements for small birds are particularly useful for obtaining precise information about flight durations and times. Such data can only be estimated very roughly by other methods, for example, based on changes in position over time as recorded by geolocator devices or based on shading and/or temperature effects recorded by light and temperature sensors (Adamik et al. 2016; Ouwehand and Both 2016).

A complete annual activity result can be presented as an actogram, where levels of activity are plotted along a daily time axis of one line per day or often double-plotted, where two consecutive days are given on a row to allow for easy inspection of both diurnal and nocturnal activities. Actograms have for a long time been used to present activity studies on migratory birds kept in cages in laboratories (e.g. Gwinner 1967). There have been successful attempts to collect activity data on migratory songbirds using radio telemetry (Zúñiga et al. 2016), but a disadvantage is that once the study bird is out of reception range, no data are obtained. Now accelerometer loggers allow us to record and present annual actograms for wild free-ranging birds.

**Fig. 1:**
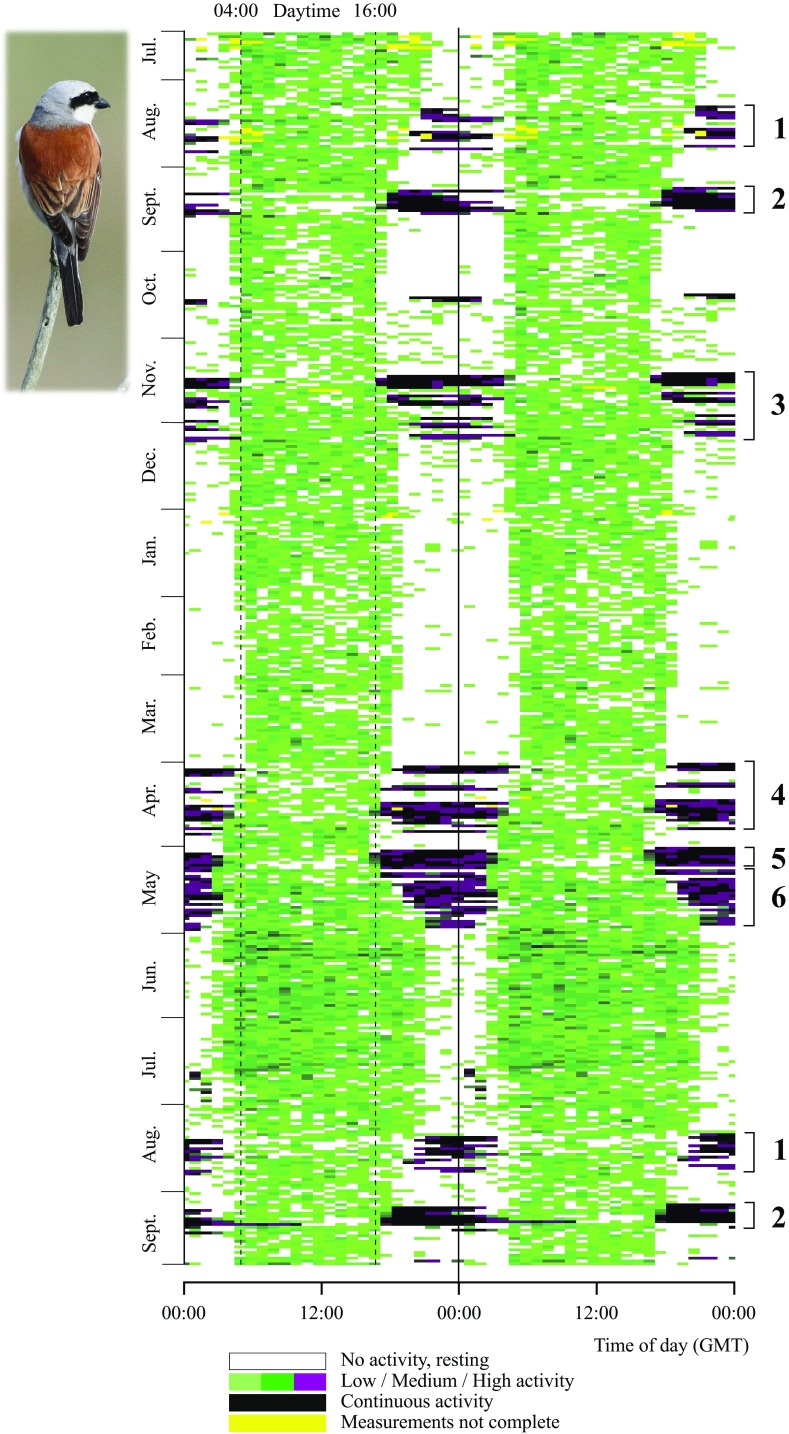
Actogram for an adult male red-backed shrike monitored form 15 July 2014 (*top*) until 27 Sep 2015 (*bottom*). *Each horizontal line* shows accelerometer data for two consecutive days, where the second day is repeated as the first day on the next line. Mean activity level was calculated for each hour ranging from 0 = no activity (*white*) to 5 = continuous activity (*black*) with intermediary levels in *colour*. Continuous and high activities (*black* and *purple*) refer to flight which occurred during night-time (with one case of prolonged flight into the succeeding day on 11 Sep 2015). Activity data were not complete (less than the expected sum of 12 activity scores per hour) during a few periods indicated in *yellow. Numbers 1*–*6* refer to different travel segments along the annual loop migration cycle (Fig. 4; Table 1 show data for the first full year cycle, while the actogram also gives information about the two first travel segments during the succeeding year)

**Fig. 2:**
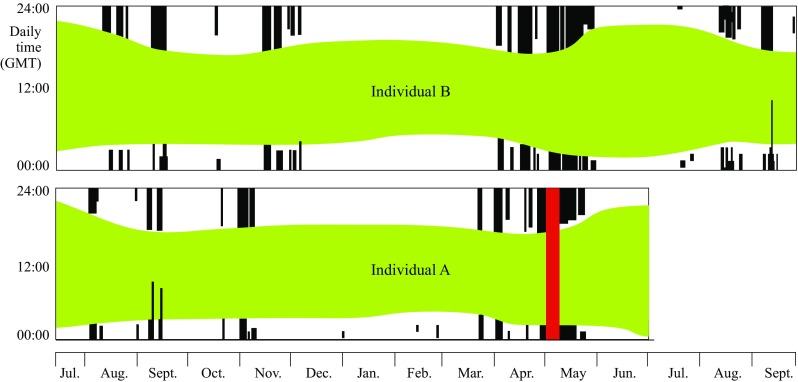
Schematic comparison of general activity patterns between the two red-backed shrikes that were recaptured in 2015 (Ind. *A, lower*) and 2016 (Ind. *B, upper*). Daily timing (hours UTC) of activities is plotted on the *y*-axis throughout the period of accelerometer measurements (*x*-axis). For detailed actograms see Bäckman et al. 2016 and Fig. 1. *Green colours* represent intermediate activity, *black* means very high (continuous flight) and *white* corresponds to no activity. The *red bar* in individual *A* indicates a 7-day period of missing data (Bäckman et al. 2016). The two individuals showed a high degree of general agreement in their actogram patterns, based on the accelerometer data, also demonstrated by a more detailed comparison of flight data in Fig. 3

**Fig. 3:**
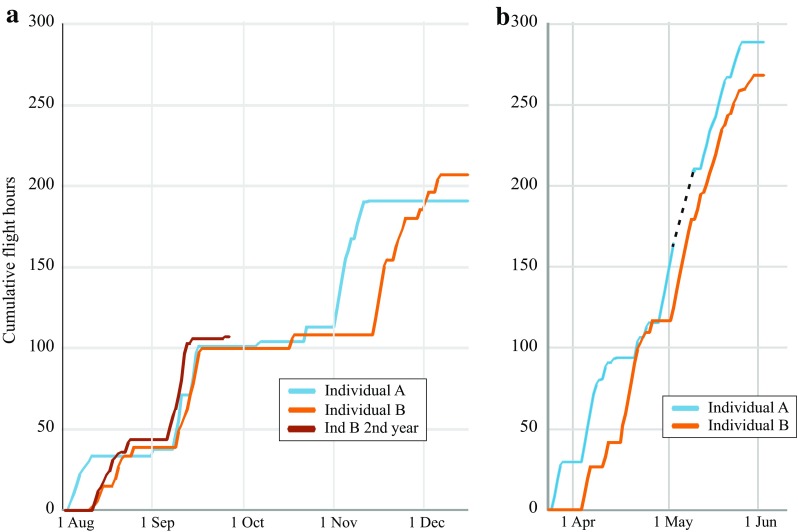
**a** Comparison of cumulative flight hours in autumn migration versus date, 1 Aug–15 Dec, and **b** comparison of cumulative flight hours in spring migration versus date, 20 Mar–1 June between the two individuals of red-backed shrike with accelerometer data. Individual *A* (Bäckman et al. 2016) used 29 flights and 191 flight hours for autumn migration and 37 flights (maximum 44 flights due to missing data for seven nights) and 243–304 flight hours for spring migration. Corresponding data for individual *B* are given in Table 1. For bird *A, broken line* indicates the addition of 7 × 6.6 h of flight during the seven nights of missing data (provisional estimate from Bäckman et al. 2016). For bird *B* flight data are not only plotted for the first year of accelerometer recording but also during the two initial travel segments of its second annual cycle involving 22 nocturnal flights and 107 flight hours (actogram in Fig. 1)

Recently, Bäckman et al. (2016) presented results from a new accelerometer datalogger that was deployed in 2014 and recovered in 2015 and used to study the annual activity of a red-backed shrike. Another red-backed shrike, carrying an identical datalogger, was retrieved in 2016 and the annual actogram of this second individual is presented in Fig. 1. The datalogger from the red-backed shrike presented in this paper continuously estimated activity levels for more than a year (14.5 months) and also provided position estimates from a few key stopover areas by using the geolocation-by-light method. In order to save energy and memory storage, diurnal light cycles were only measured for five consecutive days and the datalogger was pre-programmed with a calendar of when to run the 5-day measurement sequences. The light measurement sequences were activated on 6 occasions distributed over 1 year. The timing of measurement sequences was selected according to known staging area schedules from previously published data (Tøttrup et al. 2012a). The activity measurements ran continuously with a measurement sequence every 5 min. Each sequence consisted of 5 samples of 100 ms duration with 5 s between samples. If all samples showed no activity, the sequences scores a “0” and if all samples showed activity it scores a “5”, and intermediate cases give scores “1”–“4”. Every hour, number of scores of each value were counted and stored in a memory table that summarises activity scores during the preceding hour. The sum of score counts for each hour is always 12, which is the number of 5-min interval in an hour. Accelerometer values were collected in one axis only, approximately in parallel with gravity on a flying bird. The datalogger and its exact operation are described in full detail in Bäckman et al. (2016; see Figs. 2, 3 in that paper).

**Table 1:** Migratory flights of a red-backed shrike *Lanius collurio* as recorded by accelerometer during an annual cycle (based on the information in the actogram in Fig. 1 for the first full annual cycle)

Travel segment	Dates	Distance (km)	No. flights	Flight hours	Ground speed (km/h)	Flight duration mean (SD) (h)	Start time vs. sunset mean (SD) (h)	End time vs. sunrise mean (SD) (h)
1	11–25 Aug	1440	10	39.1	36.9	3.91 (2.02)	1.82 (0.69)	3.53 (1.90)
2	9–17 Sep	3790	9	60.7	62.4	6.75 (2.55)	1.11 (1.16)	3.59 (2.31)
3	14 Nov-6 Dec	4150	14	98.9	41.9	7.07 (2.45)	1.65 (0.97)	2.20 (1.62)
Autumn 1–3		9380	35^a^	207.3^a^	45.2			
4	2–25 Apr	4430	15	116.7	38.0	7.78 (2.59)	1.99 (1.78)	2.05 (1.54)
5	2–8 May	3220	7	62.6	51.5	8.94 (0.80)	0.76 (0.32)	0.98 (0.43)
6	10–29 May	3130	20	89.0	35.2	4.45 (2.41)	1.91 (1.46)	2.01 (1.39)
Spring 4–6		10,790	42	268.2	40.2			
Total 1–6		20,170	77^a^	475.5^a^	42.4			

**Fig. 4:**
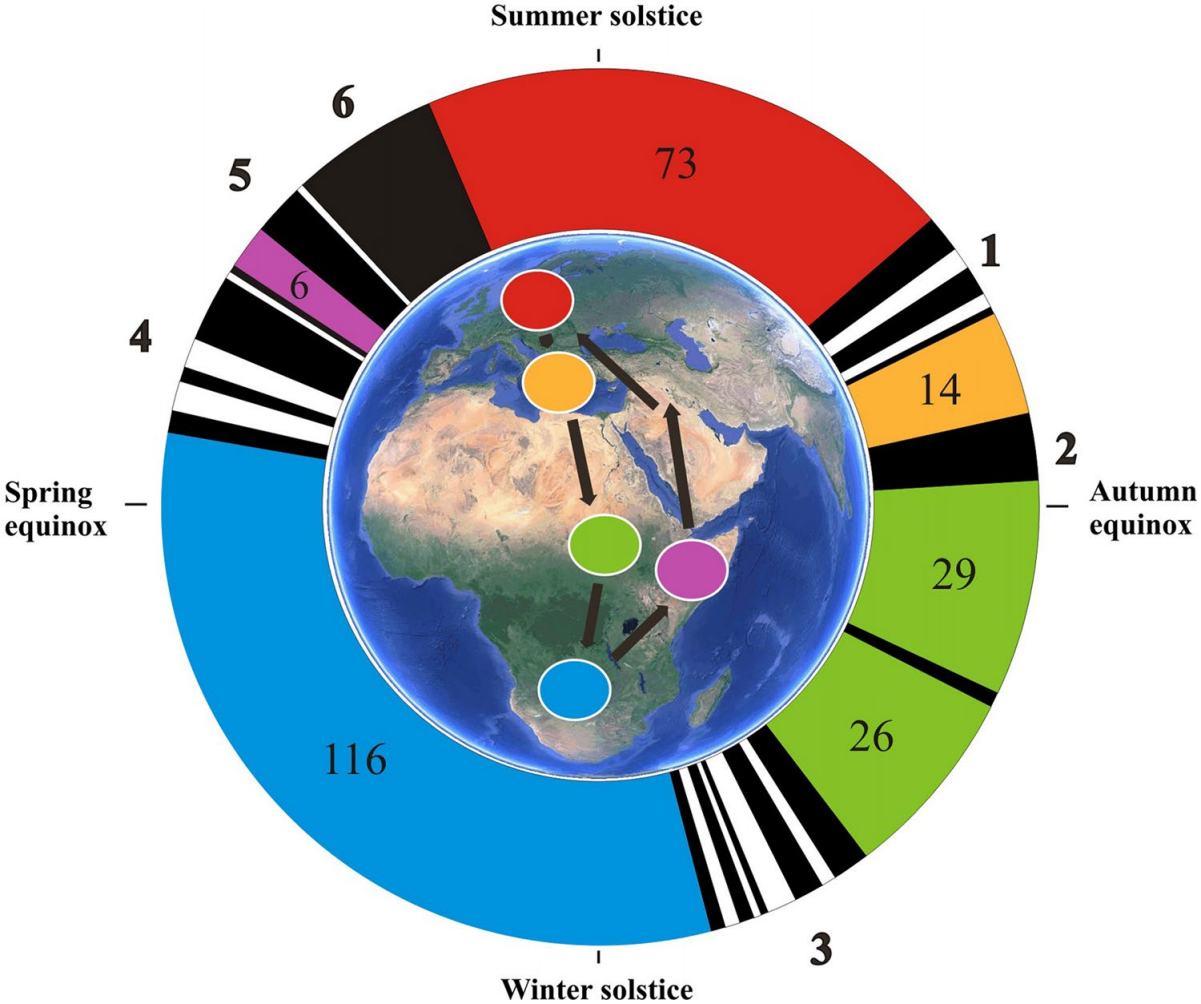
Annual cycle of red-backed shrike based on accelerometer and geolocator data. Flight activity data have been extracted from accelerometer recordings, as shown in the actogram in Fig. 1 (the initial 365 days of records for this individual). Five main stationary periods and living areas are indicated by corresponding *colours* in the circular time diagram (number of days of stay are given for each period) and on the map. *Bold numbers 1*–*6* refer to the six different travel segments as defined in Table 1, with *black* indicating days with nocturnal flights (max 20 in succession for travel segment 6; Table 1) and *white* to intervening stopover days with no nocturnal flights (max 4 in succession). The annual cycle diagram is oriented clockwise according to the annual solar cycle with summer solstice upwards (while the geographic loop migration pattern is anti-clockwise)

Travel segments refer to the main steps of migration between successive living stations in the annual cycle (Fig. 4). These travel segments were broadly defined by geolocator data for the focal individual from five periods in the year (Bäckman et al. 2016) and by geolocator data from the whole migration cycle for the study population (Tøttrup et al. 2012a). Schematic distances (rounded to nearest 10 km) refer to direct loxodrome distances of the travel segments as defined in the footnotes

Segment 1 = breeding area (56.0N, 12.3E) to SE Europe (44.0N, 20.0E), segment 2 = SE Europe (44.0N, 20.0E) to Sahel (11.0N, 30.0E), segment 3 = Sahel (11.0N, 30.0E) to S Africa (25.0S, 20.0E), segment 4 = S Africa (25.0S, 20.0E) to NE Africa (8.0N, 43.0E), segment 5 = NE Africa (8.0N, 43.0E) to Middle East (37.0N, 43.0E), segment 6 = Middle East (37.0N, 43.0E) to breeding area (56.0N, 12.3E)

^a^Two isolated nocturnal flights in October (5.25 + 3.25 h on 17 and 18 Oct)

## Migratory flights—when to fly and when to stop?

### Short and long flights

Migratory flights where birds use continuous flapping may be as short as 1–15 min or as long as 200–225 h. Short flights of only a few minutes (1–9 min covering up to 1 km) have been observed in radio-tracked Northern wheatears *Oenanthe oenanthe* during stopover at Helgoland (Schmaljohann et al. 2011). However, these flights are probably only exploratory flights to assess flight conditions and should not be classified as migratory flights. Other short flights have been indicated by radar tracking of songbirds that climb after dusk to several hundred metres of altitude only to descend steeply and land only a few kilometres away from the place of departure (own observations). Such flights presumably reflect situations where the birds encounter unfavourable winds and quickly decide to abort a flight attempt. The longest non-stop flights mentioned above have been recorded by satellite tracking of bar-tailed godwits *Limosa lapponica* flying up to 12,000 km across the Pacific Ocean during 8–9 days (Gill et al. 2009, 2014; Battley et al. 2012). Considering avian flight mechanics (Pennycuick 2008; Hedenström 2010) and the extreme physiological adaptations by the godwits (Piersma and Gill 1998; Piersma and van Gils 2011), it seems likely that the godwits’ performance is very close to the maximum non-stop duration limit for continuous flapping flight among birds. The flapping flight duration record by the godwits may even be an overestimate if they switch to gliding flight and obtain free lift from the atmospheric conditions during some parts of their long flight—this remains to be investigated by, e.g. monitoring flight altitude and wing-beat frequency by combined barometer/accelerometer logging and relate this information to meteorological conditions in the atmosphere (cf. Gill et al. 2014).

Non-stop flapping flights lasting several days and covering many thousands of kilometres have been revealed among several shorebird species by modern tracking techniques (satellite tracking, geolocators; e.g. Minton et al. 2010; Niles et al. 2010; Johnson et al. 2011; Tomkovich et al. 2013; Lindström et al. 2016; about other long barrier crossing flights see below). Current records of distances and ground speeds of such long flights may well be significantly exceeded with improved knowledge about many more migratory species (not least by using acceleration logging), because these aspects depend not only on the inherent physiological flight limits of the birds but also on wind and altitude. The fastest long-distance flights that we know about today are executed by great snipes *Gallinago media* attaining a mean ground speed of about 25 m/s (with some birds reaching 30 m/s) during non-stop flights often lasting 60–80 h between northern Europe and tropical Africa, which is much faster than for shorebird flights over the Pacific Ocean, with mean ground speeds most often below 20 m/s (Gill et al. 2009; Lindström et al. 2016).

If birds can resort to soaring flight modes during at least part of the flying time, this will open up for much longer times of staying airborne (because gliding flight is much less energy-demanding than flapping flight), even to the degree that they can live long periods of their lives on the wing. In fact, acceleration logging has been used to demonstrate the capacity of birds to stay continuously airborne for several months. Accelerometer data have shown that alpine swifts were constantly on their wing from the time they left the breeding sites in Switzerland and departed (mid-September) on migration to their tropical winter quarters in West Africa, until they returned on spring migration to the Mediterranean region (mid-April)—a period of about 7 months (Liechti et al. 2013). During a stopover period in spring in the Mediterranean region they changed from staying airborne throughout the nights to roosting on land, probably by hanging on cliffs or trees, before returning on a quick final spring migration to their breeding sites. Similar findings were recently reported also for the common swift based on acceleration logging. Adult swifts spent the entire ten-month non-breeding period mainly airborne, although with occasional bouts of nocturnal motionlessness during the winter residency period of most individuals (Hedenström et al. 2016). The whereabouts of the swifts during these bouts of immobility, which probably are associated with landings and possibly depend on shifting tropical weather conditions (Hedenström et al. 2016; sometimes with monthly/lunar rhythm indications), remain unknown. These studies confirmed the suspicion since decades that swifts may stay airborne during a large part of their annual cycle and lifetime. The alpine and common swifts showed the highest levels of flapping activity soon after sunset and again immediately prior to sunrise, which correlated with radar observations of dusk and dawn ascents among common swifts (Dokter et al. 2013; Liechti et al. 2013; Hedenström et al. 2016).

Alpine and common swifts do not start to breed until they reach an age of two or more years, and one may suspect that young birds stay airborne for a large part, perhaps even the entire pre-breeding period, which would be much longer than the above-mentioned flight periods of the adult birds. To demonstrate the flight habits of young swifts and alpine swifts (for example, by combined accelerometer and barometer logging) once they have left the nest and until they start breeding is a very difficult task, because of the low recapture probability, but hopefully not impossible. Another challenge is to understand why alpine swifts, but not common swifts, seem to switch from nocturnal roosting on the wing to hanging on cliffs or in trees during a stopover period in the Mediterranean region during spring migration. What factors would make such ground-based roosting more favourable in that particular region and situation for the alpine swifts?

Tracking radar studies have revealed how swifts that spend the nights airborne at high altitudes orient into the wind with a precision that increases with wind speed (Bäckman and Alerstam 2001). This behaviour has the effect of reducing resulting ground speed and thus mitigating displacement by wind when roosting on the wing. Even if reduced in this way, the swifts’ resulting ground speeds were still significant, with the birds moving headway when wind speed was lower than their airspeed (about 9 m/s) and being transported backwards when wind speed exceeded the airspeed (Bäckman and Alerstam 2001). Perhaps the dusk and dawn ascents is a way for the swifts to increase their visual range of landscape features and gauge their displacement during the intervening hours of darkness? It remains to be understood how the swifts (including the alpine swift) exploit rising air to their advantage during their long periods when staying airborne—which will be possible to explore when accelerometer and barometric data for individual swifts are analysed in relation to atmospheric data.

Individual great frigatebirds *Fregata minor* have also been demonstrated to stay aloft for months, based on data from GPS, ECG and accelerometer loggers (Weimerskirch et al. 2016). Here, accelerometer measurements provided information about head movements, circling or straight flight and wing-beat patterns. During 1–2 months of uninterrupted flight, the frigatebirds travelled huge distances (20,000 km or more) around the doldrums zone in the Indian Ocean using soaring flight when exploiting the turbulence (including weak thermals) below 600 metres over the sea surface. On repeated occasions they also climbed by circling in strong updrafts through clouds up to 2000 m above sea level. Using EEG recording of great frigatebirds flying over the ocean, it has recently been demonstrated how they manage to sleep during flight (Rattenborg et al. 2016; see below about sleep and flight).

Flying for long periods of time is probably not limited to swifts and frigatebirds but is likely to be revealed for other species as well, not least aerial hunters and seabirds that fly extensively not only on migration but also when foraging. Such birds may migrate in an efficient way by a fly-and-forage technique (Strandberg and Alerstam 2007; Alerstam 2011). The arctic tern *Sterna paradisaea* has the longest known annual migration distance, travelling between breeding areas in the temperate and arctic zones of the Northern Hemisphere and winter regions in the Antarctic pack-ice zone (during the austral summer). Geolocator studies have shown that, in addition to an annual migratory flight distance of about 50,000 km, the arctic terns may fly a similar distance within the staging and wintering areas that they use during the annual non-breeding period (Fijn et al. 2013; Egevang et al. 2010). However, geolocator data are not useful for demonstrating flight movements on a local scale, and accelerometer and other biologging data must be awaited for revealing the total flight time, when foraging flight time is added to the migratory transport flight times, of arctic terns and other species, and their maximum durations of uninterrupted flight.

Devices to record salt-water immersion have proven very useful to obtain ethoinformation and discriminate between foraging, flight and rest during the annual cycle of Manx shearwater *Puffinus puffinus* (Guilford et al. 2009; Freeman et al. 2013). Combining such information with accelerometer and other biologging data will reveal how the seabirds actually live their pelagic lives—how much time they spend on their wing and on the sea surface, their use of gliding vs. flapping flight, how they manage weather systems and zones with furious winds in their exposed situations over the open sea, etc.

Accelerometer data have been essential in combination with GPS, ECG and temperature recording to reveal the remarkable flight behaviour of bar-headed geese *Anser indicus* when crossing the Himalaya region (Hawkes et al. 2012; Bishop et al. 2015). The geese did not fly over the summits as earlier believed but typically they travelled through the valleys and mountain passes reaching maximum altitudes up to 5500 m a.s.l. (record of 7290 m in one case). Flight costs increased more rapidly with increasing altitude (decreasing air density) than expected, and the geese used a “roller coaster” strategy of flying closely above the underlying terrain and benefitting from orographic lift along the windward sides of ridges when possible. Flying low over the ground in valleys also brings the benefits of protection from cross- and headwinds and increasing landing opportunities (Bishop et al. 2015). Satellite tracking indicated strong constraints in climbing rates for brent geese *Branta bernicla* that climbed up to 2500–3000 m a.s.l. when crossing the Greenland ice-cap on spring migration (Gudmundsson et al. 1995). The brent geese probably proceeded up the ice slope by successive spells of flight alternating with longer resting pauses on the ice surface, but this remains to be demonstrated in more detail by future biologging studies.

### Annual rhythms of flight

The red-backed shrike illustrated in Fig. 1 was the second individual (designated as individual B) recaptured with successful annual actogram information (even including the initial two travel segments during autumn migration of the succeeding year; the actogram of the first individual A was illustrated in Bäckman et al. 2016). The highest activity levels, indicated by black and purple in Fig. 1, reflect activity that must in essence be continuous, considering the sampling scheme used (see “Accelerometer measurements and individual activity patterns in bird migration” section and Bäckman et al. 2016). We have interpreted such periods (>10 min) as periods of sustained flight. We cannot imagine any other reasonable explanation for these activity levels that occurred almost exclusively (with very few exceptions; cf. below) during the night. Thus, we regard these instances as migratory flights, which were often aggregated in travel episodes as shown in Fig. 1. We wish to stress that, although we feel quite confident that these high activity levels correspond to sustained flights, we cannot from the activity data distinguish between flights with high and low ground speeds or in different directions (we cannot exclude the possibility that some flights have been reversed/reoriented and not in the direction towards the migratory destination). Flight data were very similar for the two individuals (Figs. 2, 3). Both individuals used more flights and flight hours to complete spring compared to autumn migration. This was due to the combined effects of a longer spring than autumn migration distance in the loop migration system of the red-backed shrike and less favourable wind conditions during spring compared to autumn flights as indicated by the slower ground speed in spring (Table 1; Bäckman et al. 2016).

Extracting the occasions of migratory flights from the actogram (Fig. 1) and plotting them in a circular diagram illustrate the annual cycle of an individual songbird, as for the red-backed shrike given in Fig. 4. This individual used 35 nocturnal flights to complete its autumn migration and 42 flights were made during spring, giving a total of 77 flights to complete the annual migration round-trip of more than 20,000 km. The flight time added up to 207 h during autumn and 268 h during spring, totalling 475 h (Table 1). Such detailed quantitative information about flights and flight times provided by accelerometer data (Bäckman et al. 2016) gives unprecedented insight into the flight investment for migration among songbirds. This will make it possible to compare migratory flight investment between species, populations, age and sex groups, and also to analyse the variation between and within individuals, which could be done for different migratory segments or seasons as well as for the total annual migratory cycle.

The actogram of free-flying songbirds (Fig. 1) reveals not only number of flights and flight durations but also the detailed temporal course of migration. Comparing this information with the patterns of migratory restlessness in caged birds may help to reveal to what detail the shifts between flight activity and stationary periods are controlled by an endogenous circannual program and to what degree additional regulation from external stimuli are likely to be important. Data about migratory restlessness of caged birds are often averaged over periods of several nights, making it difficult to determine if there is an endogenous regulation of flights and staging periods on a finer time scale. Thus, the nocturnal migratory restlessness of caged red-backed shrikes (Gwinner and Biebach 1977) shows a broad seasonal agreement with the autumn and spring migration of the free-flying shrikes (Figs. 1, 2, 4) but the different well-defined travel episodes and stationary periods during autumn migration (Figs. 1, 4; Bäckman et al. 2016) seem not to be discernible from the restlessness data of caged birds. A more detailed analysis of short-term changes in intensity of restlessness of caged migrants (Berthold 1978, 1996; Gwinner and Schwabl-Benzinger 1982) is needed to reveal if there are indications of inherent control of specific travel episodes and staging events, and to determine the mechanisms involved in the day-to-day scheduling of migration including the links between circannual and circadian control of nocturnal songbird migration (Coppack and Bairlein 2011). The fact that caged songbirds often show prolonged migratory restlessness that continues well beyond the spring migration period may indicate that the circannual program provides only a sufficient reservoir of restlessness. Activity is terminated according to a set of rules based on external and internal stimuli (e.g. related to progress of migration, geographic position, food availability, condition and fuel reserves of the bird; cf. Gwinner and Czeschlik 1978, also discussing other explanation for the oversized spring migratory restlessness). Of course such a set of rules will be genetically encoded to make use of the available restlessness in a way that keeps the bird in adaptive concert with its annual migration cycle.

During its annual cycle the red-backed shrike (South Scandinavian population) exploits five main living areas—the breeding area (about 2.5 months), south-eastern Europe (0.5 months), the Sahel zone (2 months), southern Africa (4 months, where the shrikes moult flight feathers; Bruderer 2007) and eastern/north-eastern Africa (1–2 weeks) as recorded by geolocator studies (Tøttrup et al. 2012 a, b) and confirmed with more precise time resolution by accelerometer data (Fig. 4; Bäckman et al. 2016). This means that it belongs to the group of species showing itinerancy during its non-breeding life in Africa (Moreau 1972) undertaking intratropical migration between successive living quarters (Curry-Lindahl 1981; Pearson 1990; Pearson and Lack 1992; Thorup et al. 2017). Such complex strategies of itinerant non-breeding life have been revealed for an increasing number of species in the Palaearctic-African (e.g. cuckoo *Cuculus canorus*, Willemoes et al. 2014; great reed warbler *Acrocephalus arundinaceus*; Lemke et al. 2013; Kolecek et al. 2016; swift; Åkesson et al. 2012; wheatear *Oenanthe oenanthe*; Arlt et al. 2015; thrush nightingale; Thorup et al. 2017) and the Nearctic–Neotropical migration systems (veery *Catharus fuscescens*, Heckscher et al. 2015).

The stationary periods of the red-backed shrike were separated by six travel episodes/segments (Fig. 4) with aggregated nocturnal flights sometimes interspersed with a few stopover days (up to four consecutive stopover days). (Characteristics of flights during these six travel segments are given in Table 1; see also below.). The stay in the Sahel region included two days with nocturnal flights of 5.25 + 3.25 h (on 17–18 Oct) indicating that the bird moved several hundreds of kilometres, perhaps because of increasingly dry conditions, in the middle of its 2 months’ stay in this region (Fig. 4). The same type of isolated nocturnal flights in October was recorded also for the first shrike with accelerometer data (Bäckman et al. 2016), again indicating a change of residence during the Sahel stay.

Short stopover periods of up to four days were recorded during the shrike’s intratropical migration in Africa on its way south as well as north (Figs. 3, 4), which was also indicated for several individuals on the basis of geolocator data (Tøttrup et al. 2012a). The travel segment from southern to north-eastern Africa in spring seemed to vary in temporal course between the two individuals with accelerometer data, in spite of the fact that the total number of flights and flight hours was very similar for this travel segment (completed by 19 flights and 115 flight hours by the first individual and 15 flights and 117 flight hours by the second individual; Bäckman et al. 2016). Flights took place almost every night during the final spring migration across the Arabian desert (travel segment 5 in Fig. 4) and across the Middle East and Europe (segment 6). Most of the fuel reserves deposited in north-eastern Africa is probably consumed after the crossing of the Arabian desert. This would mean that the bird migrated through Europe in spring to an increasing degree as an “income migrant” (using fuel that has been deposited by daytime feeding between flights on successive nights) rather than as a “capital migrant” (using fuel reserves deposited prior to the travel episodes). This may explain why the nocturnal flights became progressively shorter during final spring migration (Table 1; see also Hedenström and Alerstam 1998; Alerstam 2003 about the time required for fuel deposition in relation to flight time).

### Diurnal rhythms of flights

**Fig. 5:**
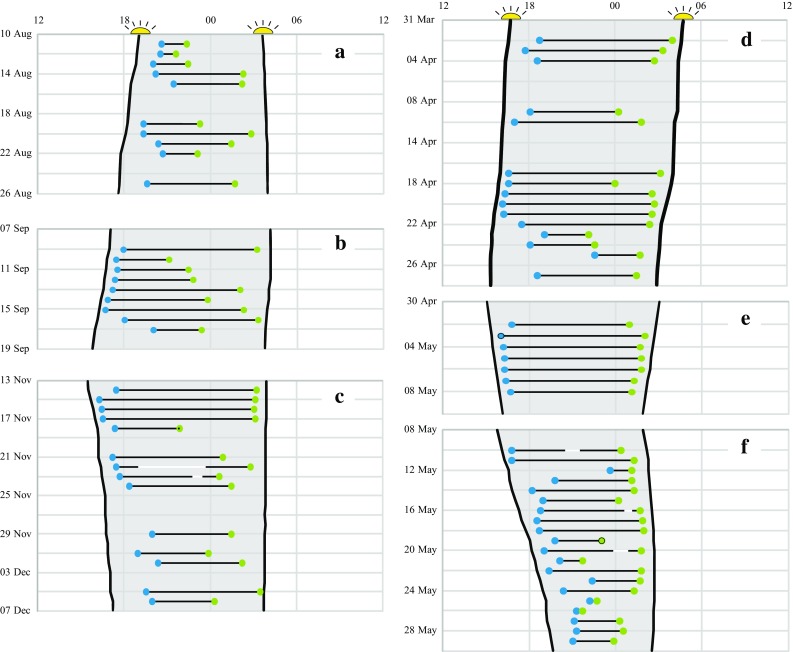
Daily timing of flights for six different travel segments during the annual cycle of a red-backed shrike based on accelerometer data (actogram in Fig. 1). Departure and landing times as well as estimated times of sunset and sunrise (nocturnal period shaded) are given in GMT (horizontal time axis from noon GMT to noon GMT the next day). Solar data were calculated from NOAA Solar Calculator (http://www.esrl.noaa.gov/gmd/grad/solcalc) for locations before and after each flight as estimated by interpolation (based on cumulative flight time) along each travel segment as defined in Table 1. Continuous flight is indicated by *black lines* (departure and landing times by *blue* and *green dots*, respectively), and interruptions, when the bird has landed to rest but departed again the same night, are indicated in *white* (five cases). **a** Flights from breeding area to SE Europe; **b** flights from SE Europe to Sahel; **c** flights from Sahel to S Africa; **d** flights from S Africa to NE Africa; **e** flights from NE Africa to Middle East; **f** flights from Middle East to breeding area (see definition of travel segments in Table 1)

The accelerometer data for the red-backed shrike (Fig. 1) allow the start and end of migratory flights to be determined with an accuracy of 5 min (Bäckman et al. 2016). These timing data can be related to the times of sunset and sunrise, provided that the location of the bird at the start and end of flights can be estimated. Such estimates of locations before and after each flight were done in a schematic way by interpolation (based on cumulative flight time) along the different main travel segments (coordinates for the start and end of each segment are given in Table 1 footnote), giving the flight times in relation to sunset and sunrise (calculated from NOAA Solar Calculator; http://www.esrl.noaa.gov/gmd/grad/solcalc) as presented in Fig. 5 and Table 1. One may fear that these schematic estimates of the birds’ locations may be too uncertain to obtain reasonable estimates of sunset/sunrise times. However, it should be noted that these times are quite robust with respect to location errors. Sunset/sunrise times are most sensitive to errors in longitude, where an error of, e.g. ±3 degrees of longitude will correspond to a sunset/sunrise timing error of only ±0.2 h. It seems likely that the geographic location estimates most often are within this level of accuracy making it possible to obtain useful estimates of flight times in relation to local sunset/sunrise. It would have been preferable to use light information to directly estimate timing of sunset/sunrise in this kind of analysis. This was not possible for the red-backed shrikes since we obtained light information from only six short periods during the year in order to minimise battery/memory use (Bäckman et al. 2016). We expect improved performance of miniature battery/memory units in the near future making direct comparisons between sunset/sunrise times (recorded by light sensor) and start/end times of flights (recorded by accelerometer) possible with a high degree of precision.

Thus, the accelerometer data for the red-backed shrike give the first glimpses of insight into the variation in flight timing within an individual throughout its annual cycle (Fig. 5; Table 1). The average departure time for this individual varied in the range of 0.7–2 h after sunset for the different travel segments (Table 1). This is in broad agreement with the take-off times for long-distance migrants during autumn in northern Europe, while short-distance migrants travelling later in the autumn season, when the nights are longer, often depart later during the night, 3–4 h after sunset (Chernetsov 2012). The accelerometer data clearly indicated the individual varied timing of flights between different travel segments, so that departures were on average earliest during barrier crossings, when the bird most often took off 0.4–0.7 h after sunset (Fig. 5b, e). Such early departures were also recorded regularly during south- and northward migration across the African continent as well as during spring migration in Europe (Fig. 5c, d, f).

Information on landing times for nocturnal passerine migrants is much more limited than on departure times. There are indications to suggest a large variability in timing of landing, with many birds landing in complete darkness in the middle of the night (Chernetsov 2012). However, there are also speculations that most migrants may not land until twilight before sunset (when they can locate preferred habitat and sites for their diurnal rest and foraging) and that duration of migratory nocturnal flights is mainly regulated by variation in departure time (Sjöberg et al. 2017). The flight data for the red-backed shrike shown in Fig. 5 did not support such reduced variability in landing compared to departure timing. On the contrary, during autumn migration landing times tended to be clearly more variable than departure times while the flights during spring migration were more symmetrical in relation to sunset and sunrise with a similar variation in starting and landing times (Fig. 5; Table 1).

The timing of departure on nocturnal flights by songbirds has been studied by visual observations (in searchlights), by capturing departing migrants in elevated mist nets as well as by radar and radio telemetry, as reviewed by Chernetsov (2012). These studies have revealed variation in departure time, with important differences between species and seasons (spring and autumn migration). Birds have been observed to depart earlier from their stopover site when night duration decreases, and parts of the differences between species and seasons can possibly be explained by differences in night duration at the period when different species migrate and between the migratory seasons. (Bolshakov et al. 2007; Schmaljohann et al. 2013; Sjöberg et al. 2017). Furthermore, the variation in departure timing has been shown to depend on condition of the bird (fuel load) and weather conditions (reviewed in Chernetsov 2012 and; Müller et al. 2016). Birds with lower fuel reserves depart later and less synchronised in time compared to individuals with larger fuel stores, possibly since their energetic reserves only last for a shorter flight, and birds delay their departures in unfavourable weather conditions (e.g. Moore and Aborn 1996; Schmaljohann and Naef-Daenzer 2011; Sjöberg et al. 2017; Smolinsky et al. 2013). These studies indicate that birds setting out on longer flights depart earlier than birds making only short flights (Mills et al. 2011; Müller et al. 2016).

The actogram results for red-backed shrike (Fig. 5) show the great potential of accelerometer data to reveal the diurnal timing of migratory flights as a basis for analyses how timing varies between species (with diurnal as well as nocturnal flight habits) and individuals as well as within individuals depending on season, remaining migratory distance, local atmospheric and astronomical conditions, etc.

### Flights across barriers

The capability among birds to undertake long non-stop flights, as discussed above (“Short and long flights” section), makes it possible for them to fly across ecological barriers, like open sea or barren desert, without landing. Such transoceanic non-stop flights have been demonstrated for shorebirds and terrestrial birds that cannot land and rest on the water surface (see above about shorebirds). For the blackpoll warbler *Setophaga striata,* it has been inferred by field and fuel deposition studies that during autumn the birds fly across the western Atlantic Ocean directly from north-eastern North America to the Caribbean region (Nisbet et al. 1995). This has been confirmed by recent geolocator studies, showing transoceanic non-stop flights lasting on average 62 h and covering 2500 km by this small songbird (DeLuca et al. 2015).

Do terrestrial birds use such non-stop flights during both day and night also when crossing inhospitable regions like deserts, where they may find shelter and rest but cannot feed? If and to what degree terrestrial migrants in the Palaearctic–African migration systems cross the Sahara (and Arabian) desert by non-stop or intermittent flight strategies have been matter of interesting debate since the suggestions of non-stop flights by Moreau (1972) were questioned in several studies reporting regular resting of songbirds during the day in the desert (Bairlein 1985, 1988; Biebach et al. 1986; Biebach 1990).

Before turning to the case of desert crossing by songbirds, it should be noted that the strategies for crossing Sahara surely differ between different categories of terrestrial bird migrants. Shorebirds are likely to cross by non-stop flights during both night and day, as exemplified by the great snipe making such non-stop flights both in autumn (when they depart from northern Europe and travel across the European continent as well as the Mediterranean Sea and Sahara desert in a single flight) and spring (Lindström et al. 2016).

Raptors and storks that travel by thermal soaring migration maintain their diurnal flight habits during the desert crossing, flying during the daily period 08–17 h when thermals are available and resting in the desert during the night waiting for the next day’s thermal period, as demonstrated by satellite and GPS tracking (e.g. Klaassen et al. 2008; Mellone et al. 2012; Vansteelant et al. 2015). Also the small hobby *Falco subbuteo*, which is less dependent on thermal soaring than larger raptors, seems to maintain its habits of diurnal flights and nocturnal resting during the Sahara crossing according to satellite tracking studies (Strandberg et al. 2009). In contrast to the large raptors, the hobbies tend to start their movement already at dawn and continue until dusk, indicating that the availability of thermals cannot be the sole explanation for their daily travel schedule—perhaps they combine migratory flight with hunting songbirds in the desert in the early morning and late afternoon.

The European roller *Coracias garrulus* is a medium-sized non-passerine trans-Sahara migrant that has been studied by satellite tracking. The results indicate that this species maintain its nocturnal migration habits when crossing the desert (Rodríguez-Ruiz et al. 2014). Perhaps this is also what the large numbers of nocturnal passerine migrants do.

Field studies in the Sahara desert during autumn demonstrated that nocturnal passerine migrants frequently landed during the day, and it was suggested that many songbirds cross the desert by a strategy of intermittent rather than non-stop flights (Bairlein 1985, 1988; Biebach et al. 1986; Biebach 1990). Both Bairlein (1985) and Biebach et al. (1986) distinguished two groups of passerine migrants that landed in the desert—(1) those with large fuel reserves that did not forage after landing but rested by sitting quietly in the shade, often in barren places without any available food and (2) individuals with reduced fuel reserves that landed in oases where they could forage during a stopover period of some days and increase their body mass and fuel reserves. Biebach (1990) pointed out that the existence of such a strategy of intermittent crossing of the desert does not exclude that non-stop flight may also be undertaken when weather (wind, temperature) permit flight with a balanced water budget.

Studies by tracking radar in the West Sahara desert (Mauretania) during autumn and spring supported the idea that an intermittent flight strategy is dominant among the passerine migrants (Schmaljohann et al. 2007). Passerine migration intensity over the desert increased abruptly at dusk indicating that birds departed from the local surroundings in the desert rather than being part of a wave of non-stop migrants passing the radar sites after having flown 24 or 48 h. The latter possibility seems to be less likely even if assumptions about the main possible departure regions for potential non-stop migrants are uncertain (Schmaljohann et al. 2007; cf.; Ouwehand and Both 2016). The radar data from the desert also demonstrated that migration intensity was reduced at dawn, indicating that many migrants landed at that time, but there was also continued migration taking place at a lower intensity, particularly in spring, during the first half of the succeeding day. This suggests that some migrants prolong flights beyond sunrise and into the next morning, perhaps on occasions when wind conditions are most favourable (Schmaljohann et al. 2007, 2009).

Recent studies by geolocators (with light as well as temperature sensors) of songbirds crossing the Sahara desert have challenged the above conclusions from field and radar studies. Adamik et al. (2016) used shading effects in the geolocator light sensor recordings to analyse daily flight patterns of four species of flycatchers and warblers when crossing the desert, concluding that prolonged flights were common, while only a small proportion of migrants flew strictly during the nights. The occurrence of non-stop flights was also suspected in some cases. Whether a bird was flying or not during the light hours of the day was judged from the absence or presence of shading effects that are recorded by the light sensor when the birds move and forage in sheltered habitat. In a similar study based on geolocator light and temperature data for pied flycatchers *Ficedula hypoleuca* crossing the West Sahara, Ouwehand and Both (2016) concluded that non-stop flights lasting 40–60 h were the most common behaviour both in autumn and spring. This was based on analyses of the changes in shading patterns in the light data and of temperature recordings during the day, with reduced temperatures being assumed to reflect flight at high and cool altitudes. In autumn, the flycatchers seemed to fly over the open sea from south-western Iberian Peninsula directly to West Africa, which would explain non-stop flights, but during spring the crossings of the flycatchers took place over the same desert regions that were surveyed by the radar studies described above.

The conclusions about the occurrence and frequency of non-stop flights, prolonged flights and strict nocturnal flights during the desert crossing are partly contradictory between these different studies. This may suggest that there exists a great diversity in flight habits among species and individuals. The crux of interpreting flight habits indirectly from light or temperature recordings is to distinguish flight from motionless resting during the day in places in the desert not exposed to direct sunlight and possibly with a cool microclimate, since no calibrations of light and temperature sensor output to possible conditions at birds’ potential resting places in the desert have been made.

**Fig. 6:**
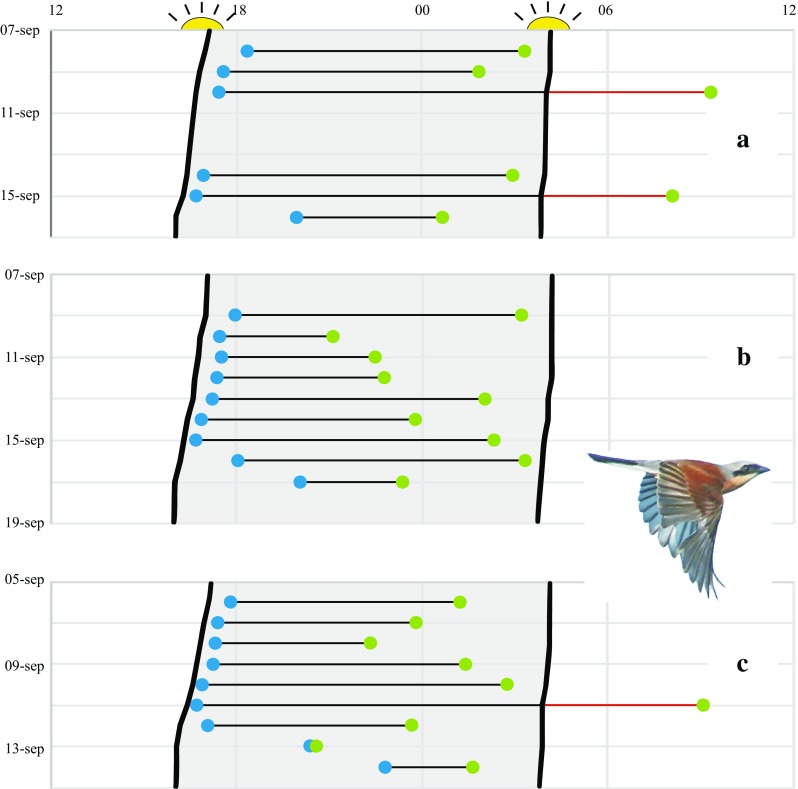
Daily timing of flights associated with the crossing of the Mediterranean Sea and the Sahara desert during the autumn migration of two individuals of red-backed shrike, as indicated by accelerometer data. This travel segment is assumed to extend from SE Europe (44.0N, 20.0E) to Sahel (11.0N, 30.0N) based on geolocator information from these two individuals as well as other individuals in the population (Tøttrup et al. 2012a). Departure (*blue dots*) and landing times (*green dots*) as well as estimated times of sunset and sunrise (nocturnal period shaded) are given in GMT (horizontal time axis from noon GMT to noon GMT the next day). Solar data were calculated from NOAA Solar Calculator (http://www.esrl.noaa.gov/gmd/grad/solcalc) for locations before and after each flight as estimated by interpolation (based on cumulative flight time) along the travel segment. Continuous flight is indicated by *black* and *red lines* (*red* when the flight is prolonged beyond sunrise and into the following day). **a** Barrier crossing by the first individual (individual *A*) in autumn 2014 (Bäckman et al. 2016). The travel segment included one initial nocturnal flight (4.5 h on 1 Sep; cf. Bäckman et al. 2016) not shown in the figure. Total flight time for the travel segment was 67.8 h (7 flights). During this crossing there was a 3-day stopover period in the Sahara desert (11–13 Sep; cf. Bäckman et al. 2016). **b** Barrier crossing by the second individual (ind. *B*) in autumn 2014. Total flight time for the travel segment was 60.7 h (9 flights). **c** Barrier crossing by the second individual (ind. *B*) in autumn 2015. Total flight time for the travel segment was 62.2 h (9 flights)

Accelerometer data provide more direct evidence of the exact timing and duration of the birds’ flights, and we can expect that desert-crossing strategies will be explored by this method among different species in the near future. The data for two individuals of red-backed shrike give a foretaste of such results (Fig. 6). Accelerometer data from three autumn crossings of the Sahara desert by these two individuals showed that strict nocturnal flights were most common (note that the travel segment included also the crossing of the Mediterranean Sea), but three cases of prolonged flights were also recorded, lasting until 4.2 and 5.3 h after sunrise (Fig. 6a, first individual in 2014; Bäckman et al. 2016) and until 5.2 h after sunrise (Fig. 6c, second individual in 2015). The first individual made a 3-day stopover (with activity indicating foraging during the 3 days) presumably at an oasis in the desert (Fig. 6a; Bäckman et al. 2016). The second individual crossed without stopover and by a succession of strictly nocturnal flights in 2014 (Fig. 6b). Both individuals were also recorded (accelerometer data) when crossing the Arabian desert in spring 2015, in both cases by an uninterrupted succession of strictly nocturnal flights (five flights ending 0.2–1.6 h before sunrise in the first individual; Bäckman et al. 2016, and seven flights ending 0.6–1.2 h before sunrise in the second individual, Fig. 5e). Hence, for the red-backed shrike, a strategy of intermittent flights across the desert is indicated, mostly by strictly nocturnal flights but occasionally with prolonged flights lasting 15.9, 15.4 and 15.8 h (the three prolonged flights in Fig. 6).

We are looking forward to future exploration of the songbirds’ fascinating desert crossing behaviour by accelerometer studies, complemented by barometric data to reveal their flight altitudes and making it possible to test the hypothesis about the association of prolonged flights (and possibly non-stop flights) and favourable flight conditions with respect to wind and temperature.

### Flights and light regimes

The accelerometer data for the red-backed shrike showed that the duration of nocturnal flights decreased as the nights became shorter when the bird migrated southwards in the Southern Hemisphere (Fig. 5c) and northwards during the Northern Hemisphere spring season (Fig. 5f). However, there was no tendency of earlier departure times after sunset with shorter night lengths as demonstrated in several studies (Chernetsov 2012; Schmaljohann et al. 2013; Sjöberg et al. 2017). One may speculate that migration towards non-breeding quarters is under less time pressure than migration towards breeding sites (Nilsson et al. 2013), making an association between short night lengths and early departures less likely for the migration towards non-breeding destinations in the Southern Hemisphere. During spring migration in the Northern Hemisphere, the shrike was flying on almost every night during the period 2–29 May (flights were conducted on 27 out of the 28 nights; cf. Fig. 5; Table 1). As a consequence, the shrike may have become an “income migrant” (see “Annual rhythms of flight” section above) with fuel reserves enough for only short nocturnal flights during the final nights of spring migration. This would mean that the association between short nights and early departures demonstrated in the above-mentioned studies is explained by species using other strategies than the shrikes during spring migration and having sufficient fuel reserves to support flights long enough to promote early departures as a result of the increased constraints on flight duration resulting from decreasing night lengths.

Many nocturnal migrants continue to breeding ranges further north than the red-backed shrike, sometimes north of the Arctic Circle. How will the changing light regimes affect these migrants as the nights become successively shorter and eventually disappear when the birds reach regions in the far north where the sun never sets? Accelerometer studies will be ideal to address this fascinating question on an individual level. Different hypotheses about nocturnal passerine migration under arctic light conditions have been discussed by Nilsson et al. (2015). Studies by tracking radar indicated that a distinct migratory peak around local midnight was still maintained when the birds travelled under continuous daylight (midnight sun) conditions, with a peak period that may have become protracted due to the lack of synchronising effects of twilight periods (Nilsson et al. 2015). Activity rhythms under polar conditions show a remarkable degree of adaptive flexibility (Lesku et al. 2012; Steiger et al. 2013), and accelerometer studies of nocturnal migrants travelling northwards during progressively shorter spring nights into the zone of continuous daylight will be awaited with greatest excitement, revealing how flight activity rhythms are controlled under different light regimes.

### Odd flights

The actogram of the red-backed shrike (Fig. 1) most often showed a total lack of indications of continuous flight activity in-between the well-defined travel segments with nocturnal flights (records of high activity levels lasting less than 5–10 min at isolated and sporadic accelerometer measurements were not regarded and included as flights; Bäckman et al. 2016). However, there were a few exceptions. The actogram in Fig. 1 showed indications of brief nocturnal flight activity, taking place after midnight, during two summer nights in 2014 (16 and 23 July, lasting 0.3–1 h) and on six summer nights during the period 20–29 July 2015 (lasting 15–30 min on each occasion). If they reflect flights in a consistent direction the bird has dispersed only a few kilometres, up to about 40 km for the longest summer flight, from the departure site. This happened more than 2 weeks prior to the first autumn migration segment when the bird travelled from the breeding region to south-eastern Europe (Table 1), so these summer flights were probably not part of any gradual transition to nocturnal migratory activity in the form of preparatory flights (cf. Zúñiga et al. 2016). They may be related to the local nocturnal movements that have been recorded among reed warblers *Acrocephalus scirpaceus* that were experimentally exposed to simulated nest predation or to short-range translocation during the summer breeding season (Mukhin et al. 2009). Hence, the shrike showing indications of short nocturnal summer flights may have suffered from breeding disturbance/failure and as a result engaged in local nocturnal movements (that may have included revisits to the breeding site; see Mukhin et al. 2009). Mukhin et al. (2009) suggested that *Acrocephalus* species, being habitat specialists, may be particularly prone to nocturnality for their local summer movements. However, the red-backed shrike is less of a habitat specialist, and this indicates that nocturnal summer movements may be more widespread among songbird species than expected. Nocturnal summer flights, possibly reflecting postfledging dispersal or navigational calibration, have also been recorded as regular among young reed warblers (Mukhin 2004; Mukhin et al. 2005).

Blackpoll warblers have been observed to perform extensive (up to 484 km) post-breeding movements before starting their migration across the Atlantic Ocean to South America (Brown and Taylor 2015). The movement of the juvenile blackpoll warblers seems to be related to regional exploration, while the movements of the adults could be correlated to the initiation of migration since their net displacement resulted in a shortening of the coming ocean crossing. However, it is not clear whether the movements by the blackpoll warblers were strictly nocturnal.

While the actogram in Fig. 1 showed a total lack of continuous flight activity during the stay in southern Africa from December until early April, the first individual with accelerometer data (actogram in Bäckman et al. 2016) carried out three nocturnal flights during the winter period (1.2 h on the night of 1 Jan, 1.0 h on 14 Feb and 2.7 h on 27 Feb), which took place after midnight and possible reflected changes in winter locations by a few tens of kilometres (or they may have been caused by nightly disturbances).

These mysterious indications of short summer and winter flights, not associated with any migratory travel segment, remain to be explored and interpreted. It is interesting that in all cases the timing of these summer and winter flights was late, after midnight, which is in clear contrast to the typical timing of migratory flights (cf. Mukhin et al. 2009).

## Rhythms of rest and activity throughout the year

### Diurnal activity patterns during a migratory life

**Fig. 7:**
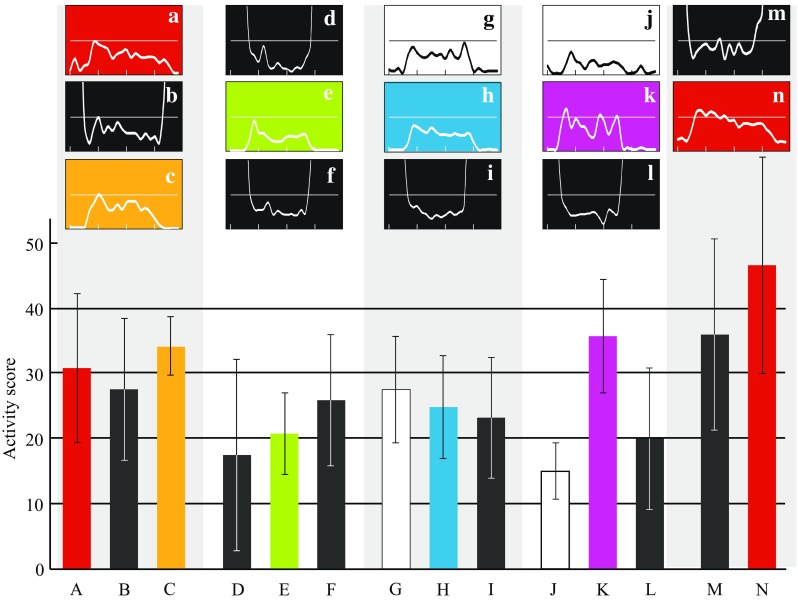
Daytime activity scores during different periods and phases of the annual cycle of a red-backed shrike, based on accelerometer data. The *histogram* shows mean daily activity scores during 12 h (04–16 h GMT, always within daytime period; see actogram in Fig. 1) for five residence periods (with *colours* corresponding to those in the annual cycle diagram in Fig. 4) and for different travel segments with *black* referring to days with migratory flights during the preceding and/or succeeding night and *white* to stopover days without migratory flights during preceding/succeeding nights. *Rectangular diagrams* show the variation in hourly activity score during the 24 h of the day (from midnight to midnight on the time axis). Periods with migratory flights (*black*) show high activity levels extending above the diagram upper limits during the dark hours, reflecting nocturnal flights. Note that the mean activity scores in the histogram refer exclusively to the daytime period 04–16 h GMT. **a** Late breeding period 15 July–10 Aug 2014, 27 days. **b** Travel segment 1 from breeding area to SE Europe, days with preceding/succeeding flights 11–26 Aug, 14 days. **c** Residence period in SE Europe 27 Aug–8 Sep, 13 days. **d** Travel segment from SE Europe to Sahel, days with preceding/succeeding flights 9–18 Sep, 10 days. **e** Residence period in Sahel 19 Sep–13 Nov, 53 days. **f** Travel segment 3 from Sahel to S Africa, days with preceding/succeeding flights 14 Nov–7 Dec, 15 days. **g** Travel segment 3 from Sahel to S Africa, stopover days without preceding/succeeding flights 11–26 Aug, 6 days. **h** Residence period in S Africa 8 Dec–1 Apr, 115 days. **i** Travel segment 4 from S Africa to NE Africa, days with preceding/succeeding flights 2–26 Apr, 19 days. **j** Travel segment 4 from S Africa to NE Africa, stopover days without preceding/succeeding flights 6–14 Apr, 6 days. **k** Residence period in NE Africa 27 Apr–1 May, 5 days. **l** Travel segment 5 from NE Africa to Middle East, days with preceding/succeeding flights 2–9 May, 6 days. **m** Travel segment 6 from Middle East to breeding area, days with preceding/succeeding flights 9–30 May, 22 days. **n** Second breeding period 31 May–11 Aug, 73 days

Accelerometer data provide information not only about numbers, timing and durations of migratory flights, but also about the daytime activity intensity during different phases of the bird’s annual life cycle. Such information will give new insights about the workload for birds using different migration strategies (cf. Flack et al. 2016), workload at different places and in different seasons as well as about differences in workload between sex and age categories. To exemplify this possibility, we illustrate the daytime activity levels and patterns for a red-backed shrike during the different periods and phases of its annual cycle (Fig. 7), based on the actogram data (Fig. 1).

The four periods with highest overall daytime activity for this individual were (1) the breeding period, (2) the period of final spring migration from the Middle East to the breeding area and the stationary periods in (3) south-eastern Europe in autumn and (4) eastern/north-eastern Africa in spring. Actually, the total sum of daytime activity during the breeding season would have been even higher compared to the other periods if the longer daily activity period during the long summer days at northern latitudes had been taken into account (see actogram in Fig. 1; the histogram in Fig. 7 refers to a 12-h daytime interval for all annual periods A-N). Considering this, the breeding season stands out as a period of very high exertion level in the life of a red-backed shrike. Comparing the breeding season activity pattern between males and females (the latter would be expected to show reduced activity in association with incubation) and among individuals in different habitats and with different reproductive outputs will be challenging tasks for the future.

The high activity level during spring migration in Europe is probably associated with a strong selection pressure to arrive early, before competitors, at the breeding destinations (Kokko 1999; Alerstam 2006; Nilsson et al. 2013). During the stationary periods in south-eastern Europe in autumn and in eastern/north-eastern Africa in spring, the shrikes are probably foraging intensively to deposit large fuel reserves in preparation for the barrier crossings (the crossing of the Mediterranean Sea/Sahara desert and of the Arabian desert, respectively).

The lowest daytime activity levels, when the bird to a high degree must have been resting during the day, were recorded for the travel days during the barrier crossings of the Mediterranean Sea/Sahara desert and Arabian Sea (Fig. 7d, l) and also for the stopover days during the bird’s spring segment from S to NE Africa (Fig. 7j). The reduced daytime activity during barrier crossings supports the view that birds landing during the day in the desert do not forage (lack of food and habitat availability) but mainly rest (see “Flights across barriers” section above). There may have been poor foraging conditions also during the bird’s spring journey from southern to north-eastern Africa, but this interpretation is less certain because activity levels were not quite so low during the travel days as during the stopover days during this segment.

The daytime pattern of activity often showed a peak in the morning, sometimes followed by a lower level of activity in the afternoon, but sometimes there were also activity peaks later in the day—as shown by the 24 h activity rhythms in the small diagrams shown in Fig. 7. Closer inspection of the activity patterns often revealed dips in activity during “quiescence” periods in the early morning, when the bird had landed after a nocturnal flight, and in the late afternoon prior to flight departure.

While the actogram data for the red-backed shrikes can only give an approximate indication of activity levels, it requires more detailed tri-axial accelerometer data to estimate energy expenditure associated with animal activity, based on calculations of the overall dynamic body acceleration (ODBA; Wilson et al. 2006; Halsey et al. 2011). In the field of bird migration, such estimates of relative energy expenditure have been made for white storks, showing that juvenile storks reduced energy expenditure when travelling across Europe with increased thermal lift and when overwintering in Africa in areas with increased human population density (associated with habitats requiring shorter foraging trips by the storks; Flack et al. 2016). Furthermore, young storks expended more energy than adults during the migratory flights, and juveniles with the highest energy expenditure showed a higher mortality compared to juveniles that were faster to learn to fly efficiently (Rotics et al. 2016).

### Flight, rest and sleep deprivation

It has been speculated for a long time that birds staying airborne during both days and nights, like swifts and frigatebirds, are able to sleep in flight and that this may possibly apply also to migratory birds that carry out long non-stop flights (Rattenborg 2006). Evidence that birds indeed do sleep in flight has recently been reported for great frigatebirds making foraging flights over the Pacific Ocean lasting an average 5.8 d (flight during day and night without landing; Rattenborg et al. 2016). Neurophysiological activity was recorded by EEG from the hyperpallium, and simultaneously accelerometer loggers recorded flight modes (wing-beat patterns, gliding and circling flight) and head movements, and GPS data loggers recorded overall movements and altitudes. This study revealed a series of exciting and remarkable results about the frigatebirds’ sleeping habits: (1) Sleep occurred much more frequently when the birds were on land (53% of total time) than in flight (only 6% of time). (2) Sleep in flight took place mainly during the night (11% of night-time but only 0.5% of daytime), while sleep was regular during both night (54% of night-time) and day (48%) when on land. (3) Sleep was more often asymmetric (difference between the cerebral hemispheres) in flight (72% of sleep time) than on land (48%). (4) Sleep was also less intense and taking place in shorter episodes in flight (average duration of sleep episodes was 14 s) than on land (52 s). (5) Sleep in flight was often associated with circling/soaring flight, with the hemisphere in the direction of circling sleeping, while the other hemisphere was awake and receiving visual information from the eye in the direction of circling. (6) Sleep intensity tended to be highest soon after landing showing a gradual decline during the hours after landing. This was interpreted as postflight recovery sleep, but it remains to be investigated if it compensates fully or not for the very limited sleep obtained in flight (Rattenborg et al. 2016).

The very fact that birds can sleep in flight is indeed an amazing finding, but it is also interesting to note the very limited extent of sleep in flight compared to when the frigatebirds were on land. This indicates that sleep in flight, even if possible, is strongly constrained and it seems unlikely that birds can satisfy their needs of sleep in flight, except perhaps birds that use gliding flight to a high degree like swifts. It is still unknown if even very small amounts of sleep can be combined with continuous flapping flight, which is the main mode of migratory flight among numerous long-distance migrants like passerines, shorebirds, waterfowl, etc.

It is an interesting question if birds that fly for many nights in succession or even non-stop during both day and night (see “Acceleration and accelerometers” section above about long flights) are affected by sleep deprivation or if there are mechanisms to mediate such effects. For the red-backed shrike, the annual migratory flights occupied a total of 440–480 h (Table 1, Bäckman et al. 2016) that otherwise would have been available for nocturnal sleep.

There seem to be at least two mechanisms that make it possible for birds to be very active during migratory periods without having their normal sleep during the dark hours of the night: (1) by increasing their resilience to sleep loss during periods of intensive activity and (2) by exploiting brief spells of compensatory sleep during daytime and immediately after long non-stop flights. Rattenborg et al. (2004) showed that nocturnal sleep loss during the migratory periods of white-crowned sparrows *Zonotrichia leucophrys* had no significant effect on the birds’ cognitive function (cage tests of the birds’ accuracy in acquisition tasks during the daytime foraging period). In contrast, there was a clear decline in task accuracy when the same birds had their nocturnal sleep experimentally disturbed during the non-migratory seasons. Birds have been observed to maintain a high level of performance in spite of serious sleep restrictions also in other situations of intensive exertion besides migratory flights, like the display period among male pectoral sandpipers *Calidris melanotos* (a 3-week period on the arctic tundra with continuous daylight conditions; Lesku et al. 2012). Males that slept the least during this display period had the highest fitness in terms of sired offspring (Lesku et al. 2012). The mechanism by which birds during such periods of intensive migratory or breeding exertion become resistant to sleep loss is unknown and it also remains to be explored what may be the costs associated with this adaptive capability of sleep suppression.

However, this mechanism seems not to allow birds to forgo sleep entirely. There are field observations to show that birds after long migratory flights have a strong urge to sleep immediately after landing (Schwilch et al. 2002). In addition, nocturnal passerine migrants like the Swainson’s thrush *Catharus ustulatus* have been demonstrated in cage studies to take brief daytime naps (unihemispheric sleep according to EEG analysis) during migration periods when their nocturnal restlessness cause extensive sleep loss (Fuchs et al. 2006, 2009). Indications of brief daytime sleep periods have also been observed in the field for migratory songbirds on stopover (Németh 2009). These evidences strongly indicate that there is still a need for at least partial sleep compensation during the birds’ migration periods, in spite of their increased resilience to sleep loss and in spite also of a possible capacity to have some limited sleep time during flapping flight (still remains to be explored; Rattenborg 2006).

We cannot find any clear indications of extensive sleep compensation before or after the different travel segments from the accelerometer data for the red-backed shrike (Fig. 1). However, on a finer time scale there are some interesting patterns. Much daytime resting took place during the crossings of the Sahara and Arabian deserts during autumn and spring, respectively (clearly seen from the actogram given in Fig. 1 as well as from the low mean daytime activity scores for travel segment 2 and 5, Fig. 7d, l). This probably reflects the fact that the birds land and rest in the desert during daytime as discussed above (section about barrier crossing flights). The main reason for this behaviour may be associated with maintaining the water balance (Biebach 1990) but the opportunity of sleep compensation may be an additional selective factor.

Daytime activity intensity was not consistently low during all travel segments (black bars in Fig. 7), indicating that compensatory sleep was more limited during travel segments across more benign regions when the birds presumably foraged mainly during daytime between the nocturnal flights. Still, early-morning and late-evening dips in activity possibly reflect post- and pre-flight compensatory sleep, and daytime activity was probably not so high as to preclude daytime micro-naps, as described above (Fuchs et al. 2006, 2009).

The most intensive daytime activity periods in the annual cycle of the red-backed shrike were the final segment of spring migration and the breeding period in June and July (Figs. 1, 7m, n). These are periods when one would expect that a mechanism providing resistance to sleep loss would be of greatest adaptive value (Rattenborg et al. 2004; Lesku et al. 2012).

Much remains to be understood about the need of sleep and how it affects birds’ migration strategies and daily activity cycles. Accelerometer measurements will be instrumental in identifying rest/sleep periods during migration and other phases of the annual cycle of free-living birds.

## Concluding remarks

Overall, the development of accelerometers has opened up new research dimensions in the study of migratory birds. Further advances in technology will allow for even smaller and more capable data loggers.

An example of technical development that will assist in designing improved accelerometer data loggers is that modern electronic sensors often include more logic in the sensor system itself, making it possible to perform some simpler operations within the sensor chip. There are new accelerometer sensors which include built-in movement detection with a configurable threshold that activates sampling operation without intervention from an external microcontroller, resulting in lower power consumption. This and other improvements will reduce the system energy consumption which translates into smaller batteries and/or more advanced operations.

Migratory birds are almost always out of sight, but trials from aviaries or field studies of breeding birds through video-recording or live observations can yield a “calibration” and validation of signatures from raw accelerometer measurements. With background data like these, we can hopefully develop energy-efficient and accurate detection algorithms for at least some key behaviour types (prey searching, attacking, prey handling), providing more detailed knowledge of behaviour behind detection of intermediate levels of activity. Combining accelerometers with supporting sensors that estimate position (GPS, geolocators, pressure sensors) or additional movement data (gyroscopes) has great potential to generate new and detailed knowledge of the migration behaviour in small birds .

## References

[CR1] Adamik P, Emmenegger T, Briedis M, Gustafsson L, Henshaw I, Krist M, Laaksonen T, Procházka P, Salewski V, Hahn S (2016). Barrier crossing in small avian migrants: individual tracking reveals prolonged nocturnal flights into the day as a common migratory strategy. Sci Rep.

[CR2] Åkesson S, Klaassen RHG, Holmgren J, Fox JW, Hedenström A (2012). Migration routes and strategies in a highly aerial migrant, the common swift *Apus apus*, revealed by light-level geolocators. PLoS ONE.

[CR3] Alerstam T, Berthold P, Gwinner E, Sonnenschein E (2003). Bird migration speed. Avian migration.

[CR4] Alerstam T (2006). Strategies for the transition to breeding in time-selected bird migration. Ardea.

[CR5] Alerstam T (2011). Optimal bird migration revisited. J Ornithol.

[CR6] Arlt D, Olsson P, Fox JW, Low M, Pärt T (2015). Prolonged stopover duration characterizes migration strategy and constraints of a long-distance migrant songbird. Anim Migr.

[CR7] Bäckman J, Alerstam T (2001). Confronting the winds: orientation and flight behaviour of roosting swifts, *Apus apus*. Proc R Soc Lond B.

[CR8] Bäckman J, Andersson A, Alerstam T, Pedersen L, Sjöberg S, Thorup K, Tøttrup A (2016). Activity and migratory flights of individual free-flying songbirds throughout the annual cycle: method and first case study. J Avian Biol.

[CR9] Bairlein F (1985). Body weights and fat deposition of Palaearctic passerine migrants in the central Sahara. Oecologia.

[CR10] Bairlein F (1988). How do migratory songbirds cross the Sahara?. TREE.

[CR11] Battley PF, Warnock N, Tibbitts TL, Gill RE, Piersma T, Hassel CJ, Douglas DC, Mulcahy DM, Gartell BD, Schuckard R, Melville DS, Riegen AC (2012). Contrasting extreme long-distance migration patterns in bar-tailed godwits *Limosa lapponica*. J Avian Biol.

[CR12] Berthold P, Schmidt-Koenig K, Keeton WT (1978). Concept of endogenous control of migration in warblers. Animal migration, navigation and homing.

[CR13] Berthold P (1996). Control of bird migration.

[CR14] Bhushan B (2010). Springer handbook of nanotechnology.

[CR15] Bidder OR, Walker JS, Jones MW, Nolton MD, Urge P, Scantlebury DM, Marks NJ, Magowan EA, Maguire IE, Wilson RP (2015). Step by step: reconstruction of terrestrial animal movement paths by dead-reckoning. Movement. Ecology.

[CR16] Biebach H, Gwinner E (1990). Strategies of trans-Sahara migrants. Bird migration. Physiology and ecophysiology.

[CR17] Biebach H, Friedrich W, Heine G (1986). Interaction of bodymass, fat, foraging and stopover period in trans-Sahara migrating passerine birds. Oecologia.

[CR18] Bishop CM, Spivey RJ, Hawkes LA, Batbayar N, Chua B, Frappell PB, Milsom WK, Natsagdorj T, Newman SH, Scott GR, Takekawa JY, Wikelski M, Butler PJ (2015). The roller coaster flight strategy of bar-headed geese conserves energy during Himalayan migrations. Science.

[CR19] Bolshakov CV, Chernetsov N, Mukhin A, Bulyuk VN, Kosarev V, Ktitorov P, Leoke D, Tsvey A (2007). Time of nocturnal departures in European robins, *Erithacus rubecula*, in relation to celestial cues, season, stopover duration and fat stores. Anim Behav.

[CR20] Bouten W, Baaij EW, Shamoun-Baranes J, Camphuysen KCJ (2013). A flexible GPS tracking system for studying bird behaviour at multiple scales. J Ornithol.

[CR21] Broell F, Noda T, Wright S, Domenici P, Steffensen JF, Auclair J-P, Taggart CT (2013). Accelerometer tags: detecting and identifying activities in fish and the effect of sampling frequency. J Exp Biol.

[CR22] Brown JM, Taylor PD (2015). Adult and hatch-year blackpoll warblers exhibit radically different regional-scale movements during post-fledging dispersal. Biol Lett.

[CR23] Brown DD, Kays R, Wikelski M, Wilson R, Klimley AP (2013). Anim Biotelem.

[CR24] Bruderer B (2007). Notes on the moult of red-backed shrikes (*Lanius collurio*) in their non-breeding range. J Ornithol.

[CR25] Chernetsov N (2012). Passerine migration. Stopover and flight.

[CR26] Collins PM, Green JA, Warwick-Evans V, Dodd S, Shaw PJA, Arnould JPY, Halsey LG (2015). Interpreting behaviors from accelerometry: a method combining simplicity and objectivity. Ecol Evol.

[CR27] Coppack T, Bairlein F (2011). Circadian control of nocturnal songbird migration. J Ornithol.

[CR28] Curry-Lindahl K (1981). Bird migration in Africa.

[CR29] Dadafshar M (2014) Accelerometer and gyroscopes sensors: operation, sensing and applications. Application note 5830. Maxim integrated systems. http://www.maximintegrated.com/en/an5830. Accessed 23 Sep 2016

[CR30] DeLuca WV, Woodworth BK, Rimmer CC, Marra PP, Taylor PD, McFarland KP, Mackenzie SA, Norris R (2015). Transoceanic migration by a 12 g songbird. Biol Lett.

[CR31] Dokter AM, Åkesson S, Beekhuis H, Bouten W, Buurma L, van Gasteren H, Holleman I (2013). Twilight ascents by common swifts, *Apus apus*, at dawn and dusk: acquisition of orientation cues?. Anim Behav.

[CR32] Egevang C, Stenhouse IJ, Phillips RA, Petersen A, Fox JW, Silk JRD (2010). Tracking of arctic terns *Sterna paradisaea* reveals longest animal migration. PNAS.

[CR33] Fijn RC, Hiemstra D, Phillips RA, van der Winden J (2013). Arctic terns *Sterna paradisaea* from the Netherlands migrate record distances across three oceans to Wilkes Land, East Antarctica. Ardea.

[CR34] Flack A, Fiedler W, Blas J, Pokrovsky I, Kaatz M, Mitropolsky M, Aghababyan K, Fakriadis I, Makrigianni E, Jerzak L, Azafzaf H, Feltrup-Azafzaf C, Rotics S, Mokotjomeal TM, Nathan R, Wikelski M (2016). Costs of migratory decisions: a comparison across eight white stork populations. Sci Adv.

[CR35] Freeman R, Dean B, Kirk H, Leonard K, Phillips RA, Perrins CM, Guilford T (2013). Predictive ethoinformatics reveals the complex migratory behaviour of a pelagic seabird, the Manx Shearwater. J R Soc Interface.

[CR36] Fuchs T, Haney A, Jechura TJ, Moore FR, Bingman VP (2006). Daytime naps in night-migrating birds: behavioral adaptation to seasonal sleep deprivation in the Swainson’s thrush, *Catharus ustulatus*. Anim Behav.

[CR37] Fuchs T, Maury D, Moore FR, Bingman VP (2009). Daytime micro-naps in a nocturnal migrant: an EEG analysis. Biol Lett.

[CR38] Gill RE, Tibbitts TL, Douglas DC, Handel CM, Mulcahy DM, Gottschalck JC, Warnock N, McCaffery BJ, Battley PF, Piersma T (2009). Extreme endurance flights by landbirds crossing the Pacific Ocean: ecological corridor rather than barrier?. Proc R Soc B.

[CR39] Gill RE, Douglas DC, Handel CM, Tibbits TL, Hufford G, Piersma T (2014). Hemispheric-scale wind selection facilitates bar-tailed godwit circum-migration of the Pacific. Anim Behav.

[CR40] Gudmundsson GA, Benvenuti S, Alerstam T, Papi F, Lilliendahl K, Åkesson S (1995). Examining the limits of flight and orientation performance: satellite tracking of brent geese migrating across the Greenland ice-cap. Proc R Soc B.

[CR41] Guilford T, Meade J, Willis J, Phillips RA, Boyle D, Roberts S, Collett M, Freeman R, Perrins CM (2009). Migration and stopover in a small pelagic seabird, the Manx Shearwater *Puffinus puffinus*: insights from machine learning. Proc R Soc B.

[CR42] Gwinner E (1967). Circannuale Periodik der Mauser und der Zugunruhe bei einem Vogel. Naturwissenschaften.

[CR43] Gwinner E, Biebach H (1977). Endogene Kontrolle der Mauser und der Zugdisposition bei südfinnischen und südfranzösischen Neuntöter (*Lanius collurio*). Vogelwarte.

[CR44] Gwinner E, Czeschlik D (1978). On the significance of spring migratory restlessness in caged birds. Oikos.

[CR45] Gwinner E, Schwabl-Benzinger I, Papi F, Wallraff HG (1982). Adaptive temporal programming of molt and migratory disposition in two closely related long-distance migrants, the pied flycatcher (*Ficedula hypoleuca*) and the collared flycatcher (*Ficedula albicollis*). Avian navigation.

[CR46] Halsey LG, Shepard ELC, Wilson RP (2011). Assessing the development and application of the accelerometry technique for estimating energy expenditure. Comp Biochem Physiol A.

[CR47] Hawkes LA, Balachandran S, Batbayar N, Butler PJ, Chua B, Douglas DC, Frappell PB, Hou Y, Milsom WK, Newman SH, Prosser DJ, Sathiyaselvam P, Scott GR, Takekawa JY, Natsagdorj T, Wikelski M, Witt MJ, Yan B, Bishop CM (2012). The paradox of extreme high-altitude migration in bar-headed geese *Anser indicus*. Proc R Soc B.

[CR48] Heckscher CM, Halley MR, Stampul PM (2015). Intratropical migration of a Nearctic-Neotropical migratory songbird (*Catharus fuscescens*) in South America with implications for migration theory. J Trop Ecol.

[CR49] Hedenström A (2010). Extreme endurance migration: what is the limit to non-stop flight?. PLoS Biol.

[CR50] Hedenström A, Alerstam T (1998). How fast can birds migrate?. J Avian Biol.

[CR51] Hedenström A, Norevik G, Warfvinge K, Andersson A, Bäckman J, Åkesson S (2016). Annual 10-month aerial life phase in the common swift *Apus apus*. Curr Biol.

[CR52] Johnson PW, Fielding L, Fox JW, Gold RS, Goodwill RH, Johnson PM (2011). Tracking the migrations of Pacific golden plovers (*Pluvialis fulva*) between Hawaii and Alaska: new insight on flight performance, breeding ground destinations, and nesting from birds carrying light level geolocators. Wader Study Group Bull.

[CR53] Kemp B, Janssen AJMW, Van Der Kamp B (1998). Body position can be monitored in 3D using miniature accelerometers and earth-magnetic field sensors. Electroencephalogr Clin Neurophysiol.

[CR54] Klaassen RHG, Strandberg R, Hake M, Alerstam T (2008). Flexibility in daily travel routines causes regional variation in bird migration speed. Behav Ecol Sociobiol.

[CR55] Kokko H (1999). Competition for early arrival in migratory birds. J Anim Ecol.

[CR56] Kolecek J, Procházka P, El-Arabany N, Tarka M, Ilieva M, Hahn S, Honza M, de la Puente J, Bermejo A, Gürsoy A, Bensch S, Zehtindijev P, Hasselquist D, Hansson B (2016). Cross-continental migratory connectivity and spatiotemporal migratory patterns in the great reed warbler. J Avian Biol.

[CR57] Lemke HW, Tarka M, Klaassen RHG, Åkesson M, Bensch S, Hasselquist D, Hansson B (2013). Annual cycle and migration strategies of a trans-Saharan migratory songbird: a geolocator study in the great reed warbler. PLoS ONE.

[CR58] Lesku JA, Rattenborg NC, Valcu M, Vyssotski AL, Kuhn S, Kuemmeth F, Heidrich W, Kempenaers B (2012). Adaptive sleep loss in polygynous pectoral sandpipers. Science.

[CR59] Liechti F, Witvliet W, Weber R, Bächler E (2013). First evidence of a 200-day non-stop flight in a bird. Nat Commun.

[CR60] Lindström Å, Alerstam T, Bahlenberg P, Ekblom R, Fox JW, Råghall J, Klaassen RHG (2016). The migration of the great snipe *Gallinago media*: intriguing variations on a grand theme. J Avian Biol.

[CR61] McKinnon E, Fraser KC, Stutchbury BJM (2013). New discoveries in landbird migration using geolocators, and a flight plan for the future. Auk.

[CR62] Mellone U, Klaassen RHG, Garcia-Ripollés C, Limiñana R, López-López P, Pavón D, Strandberg R, Urios V, Vardakis M, Alerstam T (2012). Interspecific comparison of the performance of soaring migrants in relation to morphology, meteorological conditions and migration strategies. PLoS ONE.

[CR63] Mills AM, Thurber BG, Mackenzie Sa, Taylor PD (2011). Passerines use nocturnal flights for landscape-scale movements during migration stopover. Condor.

[CR64] Minton C, Gosbell K, Johns P, Fox JW, Afanasyev V (2010). Initial results from light level geolocator trials on ruddy turnstones *Arenaria interpres* reveal unexpected migration route. Wader Study Group Bull.

[CR65] Moore F, Aborn D (1996) Time of departure by Summer Tanagers (*Piranga rubra*) from a stopover site following spring trans-gulf migration. Auk 113(4):949–952. Accessed from http://www.jstor.org/stable/4088878

[CR66] Moreau R (1972). The Palaearctic-African bird migration systems.

[CR67] Mukhin A (2004). Night movements of young reed warblers (*Acrocephalus scirpaceus*) in summer: is it postfledging dispersal?. Auk.

[CR68] Mukhin A, Grinkevich V, Helm B (2009). Under cover of darkness: nocturnal life of diurnal birds. J Biol Rhythms.

[CR69] Mukhin A, Kosarev V, Ktitorov P (2005). Nocturnal life of young songbirds well before migration. Proc R Soc B.

[CR70] Müller F, Taylor PD, Sjöberg S, Muheim R, Tsvey A, Mackenzie SA, Schmaljohann H (2016). Towards a conceptual framework for explaining variation in nocturnal departure time of songbird migrants. Mov Ecol.

[CR71] Németh Z (2009). Observation of daytime sleep-like behavior in a migratory songbird during stopover. Wilson J Ornithol.

[CR72] Newton I (2008). The migration ecology of birds.

[CR73] Niles LJ, Burger J, Porter RR, Dey AD, Minton CDT, Gonzales PM, Baker AJ, Fox JW, Gordon C (2010). First results using light level geolocators to track red knots in the Western Hemisphere show rapid and long intercontinental flights and new details on migratory pathways. Wader Study Group Bull.

[CR74] Nilsson C, Klaassen RHG, Alerstam T (2013). Differences in speed and duration of bird migration between spring and autumn. Am Nat.

[CR75] Nilsson C, Bäckman J, Karlsson H, Alerstam T (2015). Timing of nocturnal passerine migration in Arctic light conditions. Polar Biol.

[CR76] Nisbet IC, McNair DB, Post W, Williams TC (1995). Transoceanic migration of the blackpoll warbler: summary off scientific evidence and response to criticisms by Murray. J Field Ornithol.

[CR77] Noda T, Kawabata Y, Arai N, Watanabe S (2014). Animal-mounted gyroscope/accelerometer/magnetometer: In situ measurement of the movement performance of fast-start behaviour in fish. J Exp Mar Biol Ecol.

[CR78] Ouwehand J, Both C (2016). Alternate non-stop migration strategies of pied flycatchers to cross the Sahara desert. Biol Lett.

[CR79] Pearson DJ, Gwinner E (1990). Palaearctic passerine migrants in Kenya and Uganda: temporal and spatial patterns of their movements. Bird migration. Physiology and ecophysiology.

[CR80] Pearson DJ, Lack P (1992). Migration patterns and habitat use by passerine and near-passerine migrant birds in eastern Africa. Ibis.

[CR81] Pennycuick CJ (2008). Modelling the flying bird.

[CR82] Piersma T, Gill RE (1998). Guts don’t fly: small digestive organs in obese bar-tailed godwits. Auk.

[CR83] Piersma T, van Gils JA (2011). The flexible phenotype.

[CR84] Qasem L, Cardew A, Wilson A, Griffiths I, Halsey LG, Shepard ELC, Gleiss AC, Wilson R (2012). Tri-axial dynamic acceleration as a proxy for animal energy expenditure; should we be summing values or calculating the vector?. PLoS ONE.

[CR85] Rattenborg NC (2006). Do birds sleep in flight?. Naturwissenschaften.

[CR86] Rattenborg NC, Mandt BH, Obermeyer WH, Winsaurer PJ, Huber R, Wikelski M B, ca RM (2004). Migratory sleeplessness in the white-crowned sparrow (*Zonotrichia leucophrys gambelii*). PLoS Biol.

[CR87] Rattenborg NC, Voirin B, Cruz SM, Tisdale R, Dell’Omo G, Lipp HP, Wikelski M, Vyssotski AL (2016). Evidence that birds sleep in mid-flight. Nat Commun.

[CR88] Rodríguez-Ruiz J, de la Puente J, Parejo D, Valera F, Calero-Torralbo MA, Reyes-González JM, Zajkova Z, Bermejo A, Avilés JM (2014). Disentangling migratory routes and wintering grounds of Iberian near-threatened European rollers *Coracias garrulus*. PLoS ONE.

[CR89] Rotics S, Kaatz M, Resheff YS, Feldman Turjeman S, Zurell D, Sapir N, Eggers U, Flack A, Fiedler W, Jeltsch F, Wikelski M, Nathan R (2016). The challenges of the first migration: movement and behaviour of juvenile vs. adult white storks with insight regarding juvenile mortality. J Anim Ecol.

[CR90] Sakamoto KQ, Sato K, Ishizuka M, Watanuki Y, Takahashi A, Daunt F, Wanless S (2009). Can ethograms be automatically generated using body acceleration data from free-ranging birds?. PLoS ONE.

[CR91] Schmaljohann H, Naef-Daenzer B (2011). Body condition and wind support initiate the shift of migratory direction and timing of nocturnal departure in a songbird. J Anim Ecol.

[CR92] Schmaljohann H, Liechti F, Bruderer B (2007). Songbird migration across the Sahara: the non-stop hypothesis rejected!. Proc R Soc B.

[CR93] Schmaljohann H, Liechti F, Bruderer B (2009). Trans-Sahara migrants select flight altitudes to minimize energy costs rather than water loss. Behav Ecol Sociobiol.

[CR94] Schmaljohann H, Becker PJJ, Karaardic H, Liechti F, Naef-Daenzer B, Grande C (2011). Nocturnal exploratory flights, departure time, and direction in a migratory songbird. J Ornithol.

[CR95] Schmaljohann H, Korner-Nievergelt F, Naef-Daenzer B, Nagel R, Maggini I, Bulte M, Bairlein F (2013). Stopover optimization in a long-distance migrant: the role of fuel load and nocturnal take-off time in Alaskan northern wheatears (*Oenanthe oenanthe*). Front Zool.

[CR96] Schwilch R, Piersma T, Holmgren NMA, Jenni L (2002). Do migratory birds need a nap after a long non-stop flight?. Ardea.

[CR97] Shamoun-Baranes J, Bouten W, Emiel van Loon E, Meijer C, Camphuysen CJ (2016). Flap or soar? How a flight generalist responds to its aerial environment. Philos Trans R Soc B.

[CR98] Sjöberg S, Alerstam T, Åkesson S, Muheim R (2017) Ecological factors influence timing of departures in nocturnally migrating songbirds at Falsterbo, Sweden. Anim Behav (accepted)

[CR99] Smolinsky JA, Diehl RH, Radzio TA, Delaney DK, Moore FR (2013). Factors influencing the movement biology of migrant songbirds confronted with an ecological barrier. Behav Ecol Sociobiol.

[CR100] Steiger SS, Valcu M, Spoelstra K, Helm B, Wikelski M, Kempenaers B (2013). When the sun never sets: diverse activity rhythms under continuous daylight in free-living arctic-breeding birds. Proc R Soc B.

[CR101] Strandberg R, Alerstam T (2007). The strategy of fly-and-forage migration, illustrated for the osprey (*Pandion haliaetus*). Behav Ecol Sociobiol.

[CR102] Strandberg R, Klaassen RHG, Olofsson P, Alerstam T (2009). Daily travel schedules of adult Eurasian hobbies *Falco subbuteo—*variability in flight hours and migration speed along the route. Ardea.

[CR103] Thorup K, Tøttrup AP, Willemoes M, Klaassen RHG, Strandberg R, Vega ML, Dasari HP, Araújo MB, Wikelsi M, Rahbek C (2017). Resource tracking within and across continents in long-distance bird migrants. Sci Adv.

[CR104] Tomkovich PS, Porter RR, Loktionov EY, Niles LJ (2013). Pathways and staging areas of red knots *Calidris canutus rogersi* breeding in southern Chukotka. Wader Study Group Bull.

[CR105] Tøttrup AP, Klaassen RHG, Strandberg R, Thorup K, Willemoes Kristensen M, Søgard Jørgensen P, Fox J, Afanasyev V, Rahbek C, Alerstam T (2012). The annual cycle of a trans-equatorial Eurasian-African passerine migrant: different spatio-temporal strategies for autumn and spring migration. Proc R Soc B.

[CR106] Tøttrup AP, Klaassen RHG, Kristensen MW, Strandberg R, Vardanis Y, Å Lindström, Rahbek C, Alerstam T, Thorup K (2012). Drought in Africa caused delayed arrival of European songbirds. Science.

[CR107] Vansteelant WMG, Bouten W, Klaasen RHG, Koks BJ, Schlaich AE, van Diermen J, van Loon EE, Shamoun-Baranes J (2015). Regional and seasonal flight speeds of soaring migrants and the role of weather conditions at hourly and daily scales. J Avian Biol.

[CR108] Weimerskirch H, Bishop C, Jeanniard T, Prudor A, Sachs G (2016). Frigate birds track atmospheric conditions over month-long transoceanic flights. Nature.

[CR109] Weller TJ, Castle KT, Liechti F, Hein CD, Schirmacher MR, Cryan PM (2016). First direct evidence of long-distance seasonal movements and hibernation in a migratory bat. Sci Rep.

[CR110] Willemoes M, Strandberg R, Klaassen RHG, Tøttrup A, Vardanis Y, Howey PW, Thorup K, Wikelski M, Alerstam T (2014). Narrow-front loop migration in a population of the common cuckoo *Cuculus canorus*, as revealed by satellite telemetry. PLoS ONE.

[CR111] Williams HJ, Shepard ELC, Duriez O, Lambertucci SA (2015). Can accelerometry be used to distinguish between flight types in soaring birds?. Anim Biotelem.

[CR112] Wilson RP, White CR, Quintana F, Halsey LG, Liebsch N, Martin GR, Butler PJ (2006). Moving towards acceleration for estimates of activity-specific metabolic rate in free-living animals: the case of the cormorant. J Anim Ecol.

[CR113] Wilson RP, Liebsch N, Davies IM, Quintana F, Weimerskirch H, Storch S, Lucke K, Siebert U, Zankl S, Müller G, Zimmer I, Scolaro A, Campagna C, Plötz J, Bornemann H, Teilmann J, McMahon CR (2007). All at sea with animal tracks; methodological and analytical solutions for the resolution of movement. Bio-logging science: logging and relaying physical and biological data using animal-attached tags—proceedings of the 2005 international symposium on bio-logging. Science.

[CR114] Yoda K, Naito Y, Sato K, Takahashi A, Nishikawa J, Ropert-Coudert Y, Kurita M, Maho YL (2001). A new technique for monitoring the behavior of free-ranging Adélie penguins. J Exp Biol.

[CR115] Zeng H, Zhao Y (2011). Sensing movement: microsensors for body motion measurement. Sensors.

[CR116] Zúñiga D, Falconer J, Fudickar AM, Jensen W, Schmidt A, Wikelski M, Partecke J (2016). Abrupt switch to migratory night flight in a wild migratory songbird. Sci Rep.

